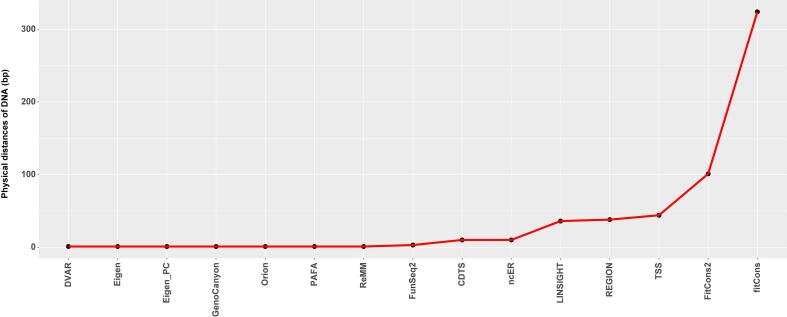# Performance Comparison of Computational Methods for the Prediction of the Function and Pathogenicity of Non-coding Variants

**DOI:** 10.1016/j.gpb.2022.02.002

**Published:** 2022-03-08

**Authors:** Zheng Wang, Guihu Zhao, Bin Li, Zhenghuan Fang, Qian Chen, Xiaomeng Wang, Tengfei Luo, Yijing Wang, Qiao Zhou, Kuokuo Li, Lu Xia, Yi Zhang, Xun Zhou, Hongxu Pan, Yuwen Zhao, Yige Wang, Lin Wang, Jifeng Guo, Beisha Tang, Kun Xia, Jinchen Li

**Affiliations:** 1 National Clinical Research Centre for Geriatric Disorders, Department of Geriatrics, Xiangya Hospital, Central South University, Changsha 410008, China; 2 Department of Neurology, Xiangya Hospital, Central South University, Changsha 410008, China; 3 Centre for Medical Genetics & Hunan Key Laboratory of Medical Genetics, School of Life Sciences, Central South University, Changsha 410008, China; 4 Reproductive Medicine Center, Xiangya Hospital, Central South University, Changsha 410008, China

**Keywords:** Non-coding variant, Pathogenicity estimation, Functional prediction, Performance assessment, Prediction model

## Abstract

**Non-coding variants** in the human genome significantly influence human traits and complex diseases via their regulation and modification effects. Hence, an increasing number of computational methods are developed to predict the effects of variants in human non-coding sequences. However, it is difficult for inexperienced users to select appropriate computational methods from dozens of available methods. To solve this issue, we assessed 12 performance metrics of 24 methods on four independent non-coding variant benchmark datasets: (1) rare germline variants from clinical relevant sequence variants (ClinVar), (2) rare somatic variants from Catalogue Of Somatic Mutations In Cancer (COSMIC), (3) common regulatory variants from curated expression quantitative trait locus (eQTL) data, and (4) disease-associated common variants from curated genome-wide association studies (GWAS). All 24 tested methods performed differently under various conditions, indicating varying strengths and weaknesses under different scenarios. Importantly, the performance of existing methods was acceptable for rare germline variants from ClinVar with the area under the receiver operating characteristic curve (AUROC) of 0.4481–0.8033 and poor for rare somatic variants from COSMIC (AUROC = 0.4984–0.7131), common regulatory variants from curated eQTL data (AUROC = 0.4837–0.6472), and disease-associated common variants from curated GWAS (AUROC = 0.4766–0.5188). We also compared the prediction performance of 24 methods for non-coding *de novo* mutations in autism spectrum disorder, and found that the combined annotation-dependent depletion (CADD) and context-dependent tolerance score (CDTS) methods showed better performance. Summarily, we assessed the performance of 24 computational methods under diverse scenarios, providing preliminary advice for proper tool selection and guiding the development of new techniques in interpreting non-coding variants.

## Introduction

Most regions of the human genome are non-coding sequences, and some of them harbor structural, regulatory, and transcribed information [1]. Some variants in non-coding sequences play important roles in human traits and complex diseases [2]. It is widely accepted that a large proportion of non-coding sequences is functional and harbors genetic variants that contribute to disease etiology [3] and that modified penetrance of pathogenic coding variants by non-coding regulatory variants can contribute to disease risk [4]. In addition, recent discoveries support that variants in non-coding sequences are important in cancer development [5], [6]. Furthermore, genome-wide association studies (GWAS) have identified numerous single-nucleotide variants (SNVs) associated with many human traits and complex diseases, and most of these associations are thought to be mediated by non-coding regulatory variants [7], [8], [9].

In the last few years, many genomic features in the non-coding sequences of the genome have been identified across multiple human tissues and cell types through various large-scale projects, such as the Encyclopedia of DNA Elements (ENCODE) [10], Roadmap Epigenomics [11], and the functional annotation of the mammalian genome (FANTOM5) [12], enabling analysis and prediction of the functional effects of non-coding variants. Several computational methods [13], [14], [15], [16], [17], [18], [19], [20], [21], [22], [23], [24], [25], [26], [27], [28], [29], [30], [31], [32] based on supervised, unsupervised, and semi-supervised models have been developed to prioritize non-coding variants by integrating various genomic features. For instance, combined annotation dependent depletion (CADD) used more than 60 various annotations from conservation, epigenetic modification, genetic context, and functional prediction [13]; Prioritization And Functional Assessment (PAFA) was the first method to introduce the fixation index [33], a population-level metric important for prioritizing population relevant functional non-coding variants [30]. Given that computational methods offer differing advantages, disadvantages, and specific features [34], users with different requirements need to choose appropriate methods. Three previous studies have evaluated the performance of several computational methods [35], [36], [37]. Nevertheless, limited benchmark datasets were used in the three studies, and they measured the area under the receiver operating characteristic (ROC) curve (AUROC) and area under the precision-recall (PR) curve (AUPRC); other critical performance metrics, such as the accuracy at 95% sensitivity or specificity, were not used. Furthermore, several recently developed methods, such as non-coding essential regulation (ncER) [28], *de novo* pattern discovery and prioritization of functional variants (DVAR) [18], and PAFA [30], have not been evaluated in detail. Hence, it is imperative to systematically and comprehensively evaluate these methods to help users choose computational methods matching their needs.

Notably, in our previous research, we did not develop any computational method for non-coding variants. Therefore, we independently assessed 12 performance metrics for 24 methods using four benchmark datasets. Our study compared computational methods under different conditions and showed that the performance of each method varied under different conditions. We also identified some computational methods with acceptable performance for rare pathogenic germline non-coding variants. We noted that no methods yielded satisfactory prediction results for rare somatic non-coding variants, disease-associated common non-coding variants, and common regulatory non-coding variants. Our results provide an opportunity for clinicians and researchers to select applicable evaluation methods to explore the functional effects of non-coding variants. Additional more accurate computational methods for various non-coding variants must be developed.

## Results

### Predictions among methods showed poor concordance

In this study, a total of 24 computational methods were assessed (Table 1). Four independent benchmark datasets were built that represented various genetic aspects: (1) rare germline variants from clinical relevant sequence variants (ClinVar), including rare non-coding germline variants of human traits and genetic diseases [38]; (2) rare somatic variants from catalogue of somatic mutations in cancer (COSMIC) for rare non-coding somatic variants of human cancers [39], [40]; (3) common regulatory variants from curated expression quantitative trait locus (eQTL) data for common non-coding variants of the human genome that explain variation in gene expression levels [41], [42], [43]; and (4) disease-associated common variants from curated GWAS for common non-coding risk variants of human diseases recognized by GWAS [43], [44] (Table 2). Further, all 24 computational methods were published before 2020 and the training datasets used were published before 2019. To reduce overlap between our testing benchmark data and the training data used in the 24 computational methods, we selected variants published after 2019 and removed variants that existed in these publicly available training datasets before comparing the methods.

**Table 1:** **Summary of 24 computational methods compared in this study**

**Method**	**Prediction model**	**Model type**	**Learning dataset**	**Version**	**Ref.**
CADD	SVM and logistic regression model	Supervised	Simulated DNMs and variants arisen and fixed in human populations	v1.3	[13]
CDTS	Difference between expected and observed score as context-dependent tolerance score	Unsupervised	11,257 human whole-genome sequences	2017	[14]
CScape	Kernel-based models and leave-one-concentration-out cross validation	Supervised	Somatic point variants from the COSMIC and SNVs from the 1000 Genomes Project	2017	[15]
DANN	Deep neural network	Supervised	Simulated DNMs and variants arisen and fixed in human populations	2015	[16]
DIVAN_TSS	Ensemble learning framework	Supervised	Risk variants of 45 diseases/phenotypes (ARB) and benign variants are sampled from the 1000 Genomes Project with TSS-matched criterion	2016	[17]
DIVAN_REGION	Ensemble learning framework	Supervised	Risk variants of 45 diseases/phenotypes (ARB) and benign variants are sampled from the 1000 Genomes Project with region-matched criterion	2016	[17]
DVAR	Multivariate Dirichlet Process Mixtures	Unsupervised	2 million variants randomly sampled from the 1000 Genomes Project	v1.0	[18]
Eigen	Spectral meta-learner	Unsupervised	Variants in the 1000 Genomes Project without a match in dbNSFP and within 500 bp upstream of the TSS	v1.1	[19]
Eigen_PC	Spectral meta-learner	Unsupervised	Variants in the 1000 Genomes Project without a match in dbNSFP and within 500 bp upstream of the TSS	v1.1	[19]
FATHMM-MKL	Multiple kernel learning	Supervised	Germline variants in HGMD and control variants from the 1000 Genomes Project	2017	[20]
FATHMM-XF	Kernel-based models and platt scaling	Supervised	Positive variants from the HGMD and control variants from the 1000 Genomes Project	2017	[21]
FIRE	Random forest model	Supervised	*Cis*-eQTL SNVs identified by the Geuvadis lymphoblastoid cell lines and sampled non-eQTL SNVs	2017	[22]
fitCons	Generative probabilistic model	Semi-supervised	Multiple species genomic DNA sequence	v1.01	[23]
FitCons2	Probabilistic evolutionary model	Semi-supervised	Multiple species genomic DNA sequence	2017	[24]
FunSeq2	Weighted scoring scheme	Semi-supervised	Small-scale informative data context from the 1000 Genomes Project, ENCODE, COSMIC, and CGC	v2.1.6	[25]
GenoCanyon	Conditional joint density estimation	Unsupervised	Each location in the human genome	v1.0.3	[26]
LINSIGHT	Combination of generalized linear model and probabilistic model	Semi-supervised	Multiple species genomic DNA sequence and 54 unrelated human genomes	2017	[27]
ncER	XGBoost model	Supervised	Positive examples from HGMD (2016_R1) and ClinVar (July 2016) and negative examples from gnomAD	v1.0	[28]
Orion	Difference between the observed and expected site-frequency spectrums	Unsupervised	1662 WGS samples	2017	[29]
PAFA	Logistic regression with L1 regularization	Supervised	Variants labeled “pathogenic” in ClinVar and significant SNPs associated with complex traits or diseases and variants labeled “benign” in ClinVar and variants in the 1000 Genomes Project	2018	[30]
regBase_REG	XGBoost model	Supervised	Functional regulatory variant dataset and non-coding variants from the 1000 Genomes Project	v1.0	[31]
regBase_PAT	XGBoost model	Supervised	Pathogenic regulatory variant dataset and non-coding benign variants labeled “benign” in ClinVar	v1.0	[31]
regBase_CAN	XGBoost model	Supervised	Cancer recurrent regulatory somatic mutation dataset and non-recurrent somatic mutations	v1.0	[31]
ReMM	Random forest model	Supervised	Hand-curated set of regulatory mendelian mutations and derived alleles of human evolution	v0.3.1	[32]

*Note*: The difference between DIVAN_TSS and DIVAN_REGION was the criteria to choose benign variants in the training set. Eigen_PC had the same prediction model and learning dataset as Eigen but they had different weights for some genomic features. regBase trained three composite models based on different training datasets to score functional, pathogenic, and cancer driver non-coding regulatory variants, respectively. CADD, combined annotation dependent depletion; CDTS, context-dependent tolerance score; DANN, deleterious annotation of genetic variants using neural networks; DIVAN, DIsease-specific Variant ANnotation; FIRE, Functional Inference of Regulators of Expression; fitCons, fitness consequences of functional annotation; ncER, non-coding essential regulation; PAFA, Prioritization And Functional Assessment; ReMM, Regulatory Mendelian Mutation; DNM, *de novo* mutation; COSMIC, the Catalogue of Somatic Mutations in Cancer; SNV, single-nucleotide variant; ARB, association results browser; TSS, transcription start site; HGMD, Human Gene Mutation Database; eQTL, expression quantitative trait locus; ENCODE, Encyclopedia of DNA Elements; CGC, Cancer Gene Census; gnomAD, Genome Aggregation Database; WGS, whole-genome sequencing; SNP, single-nucleotide polymorphism; SVM, support vector machine; XGBoost, extreme gradient boosting.

**Table 2:** **Summary of four independent benchmark datasets used in this study**

**Benchmark dataset**	**Positive set**	**Negative set**	**No. of positive variants**	**No. of negative variants**	**Refs.**
Rare germline variants from ClinVar	Non-coding ‘pathogenic’ and ‘likely pathogenic’ germline variants from ClinVar (20190102–20201128)	Non-coding ‘benign’ germline variants from ClinVar (20190102–20201128)	515	1850	[38]
Rare somatic variants from COSMIC	Non-coding somatic variants from COSMIC (v88–v92) with recurrence ≥ 2 and located on risk genes collected by CNCDatabase	Non-coding somatic variants from COSMIC (v88–v92) with recurrence = 1 and located on genes except for risk genes collected by CNCDatabase	1966	597,221	[39], [40]
Common regulatory variants from curated eQTL data	eQTL SNPs from the GTEx portal database and Brown’s study	Randomly selecting variants with matched properties from the 1000 Genomes Project by vSampler	13,274	13,274	[41], [42], [43]
Disease-associated common variants from curated GWAS	Non-coding SNVs in the intersection set of credible sets defined by three tools from CAUSALdb database with MAF > 5%	Non-coding SNVs from the 1000 Genomes Project with MAF > 5% in the same LD blocks as corresponding positive variants with r^2^ threshold > 0.2	73,693	76,214	[43], [44]

*Note*: Matched properties including MAF, distance to closet transcription start site, gene density, and number of variants in LD. Three tools include PAINTOR [62], CAVIARBF [63], and FINEMAP [64]. CNCDatabase, Cornell Non-coding Cancer driver Database; GTEx, Genotype-Tissue Expression; MAF, minor allele frequency; LD, linkage disequilibrium.

**Supplementary Figure S1:**
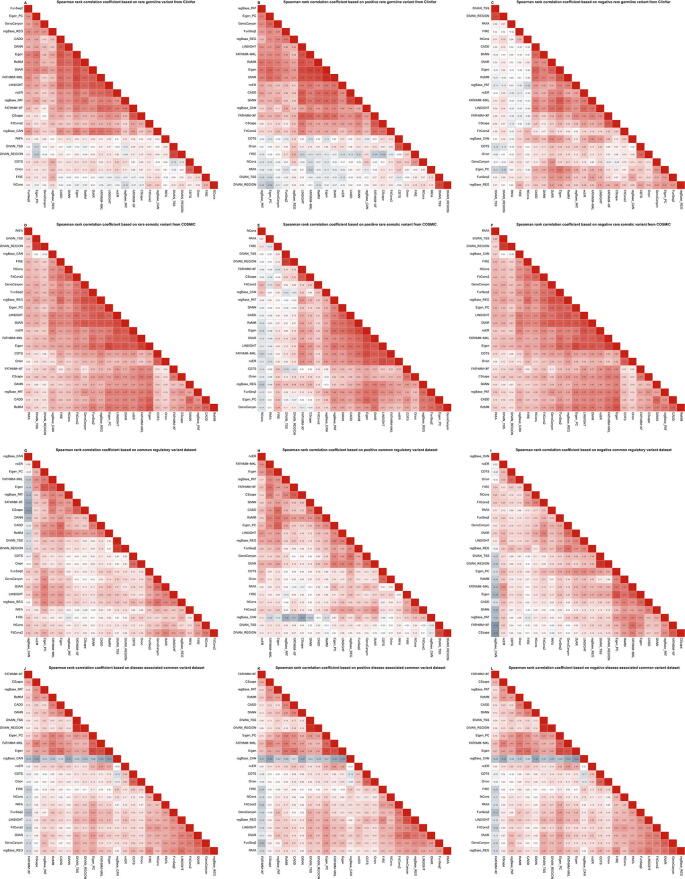


Spearman rank correlation coefficients were calculated between pairs of computational methods based on the PHRED-scaled scores of four benchmark datasets to evaluate the predictive concordances among the 24 computational methods (Figure S1). The overall pairwise correlation for rare somatic variants from COSMIC was generally higher than for the other three datasets, suggesting that current methods show better concordance in somatic variants prediction. Moreover, we calculated the Spearman rank correlation coefficient based on the positive variant dataset and negative variant dataset for each benchmark dataset. We found that the overall pairwise correlation for negative rare somatic variants from COSMIC was higher than for positive rare somatic variants from COSMIC. The weak pairwise correlations (R < 0.4) among all 24 computational methods were common in the four benchmark datasets, except for a few computational methods that were highly correlated with each other (R > 0.8) in the positive rare germline variants from ClinVar, such as CADD and deleterious annotation of genetic variants using neural networks (DANN), possibly because of the selection of similar training data and learning features. In summary, our results indicate that existing computational methods have poor predictive concordance for the same benchmark dataset, suggesting the necessity and importance of assessing different computational methods under various conditions.

### Methods showed different performances for rare germline and somatic variants

It is widely accepted that pathogenic variants are often rare variants. To determine the performance of all 24 methods for rare variants, we constructed two datasets, including rare germline variants from ClinVar and rare somatic variants from COSMIC. (1) Rare germline variants from ClinVar included 515 positive and 1850 negative variants (Table 2, Table S1), which were downloaded from ‘pathogenic’, ‘likely pathogenic’, and ‘benign’ non-coding germline variants in the ClinVar database [38] with allele frequency (AF) < 0.1% in the Genome Aggregation Database (gnomAD) [45]. (2) Rare somatic variants from COSMIC included 1966 positive and 597,221 negative variants (Table 2, Table S1), and all of these variants were downloaded from the COSMIC database [39] with AF < 0.1% in the gnomAD database. In addition, we selected AUROC as our major performance measure because, compared to other metrics, its value is unaffected by different cutoff values.

**Supplementary Figure S2:**
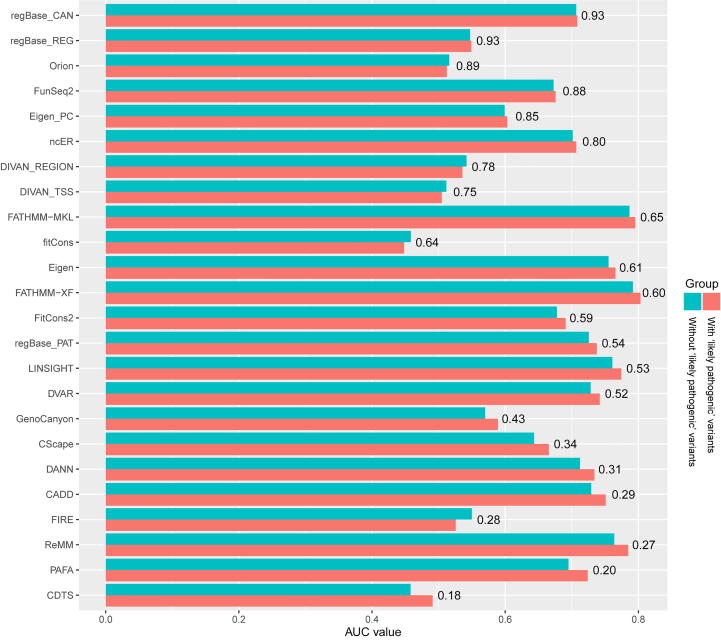


Assessments of 12 performance metrics for all 24 computational methods based on the PHRED-scaled scores of rare germline variants from ClinVar are summarized in Table 3. We found that the AUROC of the 24 methods ranged from 0.4481 to 0.8033 (median of AUROC = 0.6988), and that Functional Analysis Through Hidden Markov Models with an eXtended Feature set (FATHMM-XF [21]; AUROC = 0.8033) exhibited the best performance, followed closely by Functional Analysis Through Hidden Markov Models with multiple kernel learning (FATHMM-MKL [20]; AUROC = 0.7954) and Regulatory Mendelian Mutation (ReMM; AUROC = 0.7848). Clinicians and researchers sometimes require computational methods with high sensitivity or specificity (typically > 95%). For example, doctors may choose computational methods with high sensitivity to evaluate the pathogenicity of non-coding variants in genetic counseling for known pathogenic genes. We further assessed the high-specificity regional AUROC (hspr-AUROC) and high-sensitivity regional AUROC (hser-AUROC) values. We found that FATHMM-XF (hspr-AUROC = 0.7067) exhibited the best performance with hspr-AUROC values > 0.70, while regBase_PAT [31] (hser-AUROC = 0.5517) exhibited the best performance with hser-AUROC values > 0.55 (Table 3). The accuracy and Mathews correlation coefficient (MCC) were also used to assess the performance of computational methods, with FATHMM-XF showing the highest accuracy and MCC scores among the 24 methods. Notably, methods based on supervised models (median of AUROC = 0.7161) showed better performance than those based on semi-supervised models (median of AUROC = 0.6832) and methods based on unsupervised models (median of AUROC = 0.5961). Moreover, we assessed the performance of the 24 computational methods based on rare germline variants from ClinVar after removing the ‘likely pathogenic’ non-coding germline variants, resulting in 343 positive variants and 1850 negative variants. The assessment results of 12 performance metrics for all 24 computational methods are summarized in Table S2. Performance metrics such as the AUROC of the computational methods were generally concordant, regardless of whether the variants were likely pathogenic (Figure S2).

**Table 3:** **Performance evaluation based on rare germline variants from ClinVar**

**Method**	**Missing rate (%)**	**Best threshold**	**PPV (%)**	**NPV (%)**	**FNR (%)**	**Sensitivity (%)**	**FPR (%)**	**Specificity (%)**	**Accuracy (%)**	**MCC**	**AUROC**	**hspr-AUROC**	**hser-AUROC**	**Prediction model**
CADD	0.00	11.1395	44.40	87.83	39.22	60.78	21.19	78.81	74.88	0.3572	0.7509	0.5587	0.5277	Supervised
CScape	9.77	30.6855	37.46	85.79	48.43	51.57	22.75	77.25	71.88	0.2589	0.6655	0.5344	0.5217	Supervised
DANN	0.00	9.5376	45.95	86.78	44.85	55.15	18.05	81.95	76.11	0.3484	0.7341	0.5956	0.5244	Supervised
DIVAN_REGION	0.00	2.6953	23.89	82.34	27.57	72.43	64.22	35.78	43.76	0.0715	0.5357	0.5153	0.5064	Supervised
DIVAN_TSS	0.00	3.8817	23.16	80.52	33.59	66.41	61.35	38.65	44.69	0.0431	0.5047	0.5040	0.5028	Supervised
FATHMM-MKL	0.00	12.1444	49.10	90.16	31.46	68.54	19.78	80.22	77.67	**0.4375**	**0.7954**	**0.6359**	0.5344	Supervised
FATHMM-XF	9.77	26.0395	**60.32**	**90.98**	33.18	66.82	**11.61**	**88.39**	**83.88**	**0.5322**	**0.8033**	**0.7067**	0.5074	Supervised
FIRE	0.00	9.7388	26.09	80.95	53.59	46.41	36.59	63.41	59.70	0.0831	0.5256	NA	0.5034	Supervised
ncER	0.25	13.9272	39.92	87.50	37.94	62.06	26.02	73.98	71.39	0.3144	0.7067	0.5249	0.5161	Supervised
PAFA	8.03	1.1395	36.40	90.42	26.32	73.68	34.15	65.85	67.49	0.3256	0.7239	0.5208	NA	Supervised
regBase_CAN	0.00	10.2935	39.50	89.49	29.51	70.49	30.05	69.95	70.06	0.3423	0.7083	0.5176	0.5018	Supervised
regBase_PAT	0.00	7.3824	35.23	89.40	26.60	73.40	37.57	62.43	64.82	0.2970	0.7375	0.5721	**0.5517**	Supervised
regBase_REG	0.00	20.6843	27.53	80.37	65.63	34.37	25.19	74.81	66.00	0.0852	0.5491	NA	NA	Supervised
ReMM	0.00	13.8161	47.68	89.36	34.17	65.83	20.11	79.89	76.83	0.4115	**0.7848**	0.5969	**0.5448**	Supervised
CDTS	8.25	10.9826	25.36	79.56	66.88	33.12	27.24	72.76	64.10	0.0538	0.4910	NA	NA	Unsupervised
DVAR	0.00	15.0531	**51.63**	88.69	38.45	61.55	**16.05**	**83.95**	**79.07**	0.4283	0.7420	0.5371	0.5159	Unsupervised
Eigen	10.40	15.3901	43.68	89.58	35.48	64.52	21.42	78.58	75.70	0.3786	0.7656	0.5379	**0.5425**	Unsupervised
Eigen_PC	10.40	8.8690	27.96	88.79	**24.42**	**75.58**	50.15	49.85	55.12	0.2064	0.6032	NA	0.5366	Unsupervised
GenoCanyon	0.00	12.2531	33.39	82.57	58.25	41.75	23.19	76.81	69.18	0.1721	0.5890	0.5418	0.5012	Unsupervised
Orion	14.80	11.1831	23.61	80.75	67.07	32.93	27.47	72.53	64.42	0.0488	0.5124	0.5074	NA	Unsupervised
fitCons	8.16	0.2856	21.12	**95.45**	**0.22**	**99.78**	98.78	1.22	21.87	0.0408	0.4481	NA	0.5034	Semi-supervised
FitCons2	8.03	17.3066	41.81	86.30	48.46	51.54	19.02	80.98	74.80	0.3023	0.6909	0.6052	0.5029	Semi-supervised
FunSeq2	1.61	7.6079	29.59	**91.72**	**14.03**	**85.97**	56.84	43.16	52.47	0.2491	0.6756	0.5330	0.5299	Semi-supervised
LINSIGHT	1.82	17.6486	**64.97**	88.15	44.44	55.56	**8.31**	**91.69**	**83.85**	**0.5010**	0.7743	**0.6307**	0.5005	Semi-supervised

*Note*: Best threshold indicates the threshold corresponding to the best sum of sensitivity and specificity. Top three methods of every measure are represented by bold text. PPV, positive predictive value; NPV, negative predictive value; FPR, false-positive rate; FNR, false-negative rate; MCC, Mathew correlation coefficient; AUROC, area under the receiver operating characteristic curve; hspr-AUROC, high-specificity regional area under the receiver operating characteristic curve; hser-AUROC, high-sensitivity regional area under the receiver operating characteristic curve; NA, not available.

In addition, we assessed the performance of 24 methods for somatic variants and assessments of 12 performance metrics based on PHRED-scaled scores, as summarized in Table S3. The AUROC of the 24 computational methods ranged from 0.4984 to 0.7131 (median of AUROC = 0.6295) in rare somatic variants from COSMIC, with FunSeq2 [25] (AUROC = 0.7131) exhibiting the best overall performance, followed closely by fitness consequences 2 (FitCons2) [24] (AUROC = 0.7069). This result suggests that existing methods perform poorly for non-coding somatic variants. Furthermore, methods based on semi-supervised models (median of AUROC = 0.6988) performed better than methods based on unsupervised (median of AUROC = 0.6551) and supervised (median of AUROC = 0.6063) models.

### Predictive ability of methods for common variants warrants improvement

**Supplementary Figure S3:**
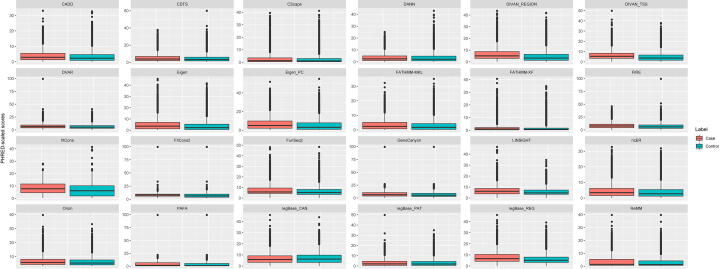


**Supplementary Figure S4:**
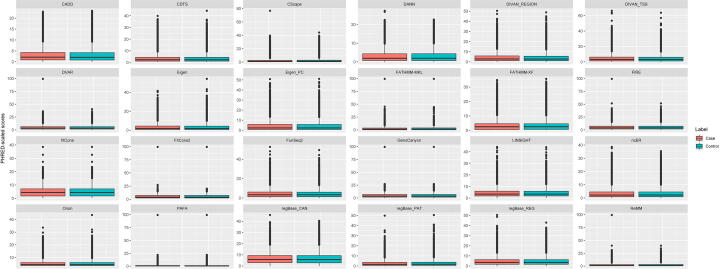


**Supplementary Figure S5:**
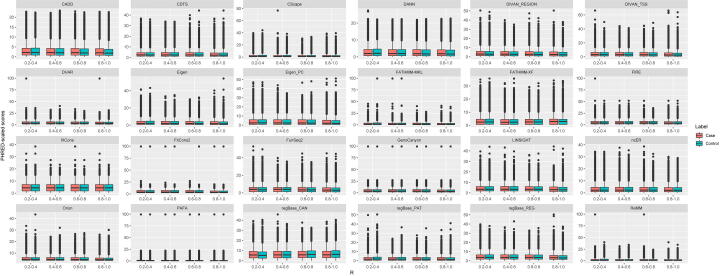


It is now accepted that some common variants are regulatory or risk variants; hence, we also constructed common regulatory variants from curated eQTL data and disease-associated common variants from curated GWAS (see Materials and methods) to evaluate the performance of 24 methods for variants in the 1000 Genomes Project [43] with AF > 5% (Table 2, Table S1). The respective numbers of positive and negative variants were recorded in the common regulatory variants from curated eQTL data (13,274 and 13,274) and disease-associated common variants from curated GWAS (73,693 and 76,214). We found that the AUROC of the 24 computational methods ranged from 0.4837 to 0.6472 (median of AUROC = 0.5619) in common regulatory variants from curated eQTL data and from 0.4766 to 0.5188 (median of AUROC = 0.5041) in disease-associated common variants from curated GWAS (Tables S4 and S5), and that the distributions of PHRED-scaled scores for positive and negative variants were similar irrespective of them being in common regulatory variants from curated eQTL data or disease-associated common variants from curated GWAS (Figures S3 and S4). This indicates that existing methods are unsuitable for common variants, particularly for common variants in the same linkage disequilibrium (LD) block. Furthermore, we classified the disease-associated common variants from curated GWAS into four subgroups (0.2–0.4, 0.4–0.6, 0.6–0.8, and 0.8–1.0) according to r^2^ thresholds of LD, and found that all methods showed poor performance for four subgroups (Figure S5).

### CADD and context-dependent tolerance score showed better performance for non-coding *de novo* mutations in autism spectrum disorder

**Figure 1:**
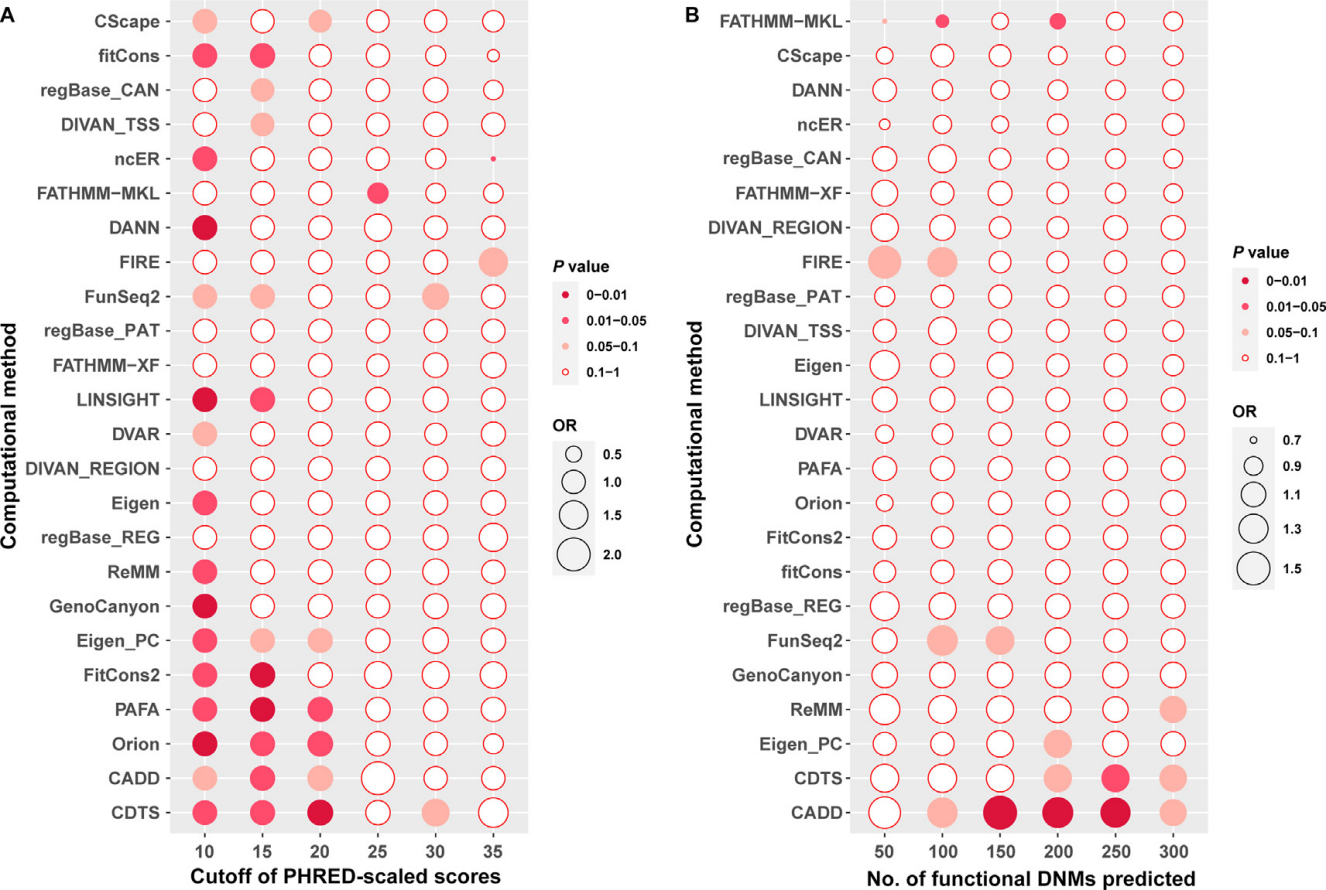
**Performance evaluation in ASD based on DNMs** **A.** Performance evaluation of 24 computational methods under different cutoff values of PHRED-scaled scores. The order of the 24 computational methods shown on the Y-axis is based on their OR values under cutoff = 20. **B.** Performance evaluation of 24 computational methods under different numbers of DNMs that are most likely to be functional in ASD. The order of the 24 computational methods shown on the Y-axis is based on their OR values with the number of most likely functional DNMs being 200. The OR and *P* values were calculated by a two-sided Poisson’s ratio test. The size of each ball is proportional to the OR value. Differently colored balls represent different *P* value ranges. OR, odds ratios; DNM, *de novo* mutation; ASD, autism spectrum disorder.

Non-coding *de novo* mutations (DNMs) play important roles in neurodevelopmental disorders [46], such as DNMs in the promoter and regulatory regions in autism spectrum disorder (ASD) [47], [48]. We then downloaded 115,569 and 113,530 non-coding DNMs from 1902 patients with ASD and 1902 unaffected siblings from the Gene4Denovo database [49], and evaluated the performance of the methods based on their PHRED-scaled scores (Figure 1; Table S6). Given that the pathogenicity of most non-coding DNMs is unclear, we selected odds ratios (OR) to assess the performance of the computational methods; better methods were expected to show higher OR under the same conditions. We adopted two strategies to calculate the OR and two-sided *P* values between patients with ASD and their unaffected siblings.

In the first strategy, we counted the number of positive non-coding DNMs in the ASD and sibling groups under different cutoff values of PHRED-scaled scores (*i.e.*, 10, 15, 20, 25, 30, and 35) for the 24 computational methods. The number of positive DNMs predicted by most methods between the ASD and sibling groups showed significant differences (*P* < 0.05) under the most relaxed condition (cutoff = 10) but had low OR (OR < 1.05). Under increasingly rigorous thresholds, many methods showed higher OR but with *P* > 0.05; the context-dependent tolerance score (CDTS) method achieved the best performance at a cutoff value of 20 (OR = 1.13, *P* = 0.006).

In the second strategy, we selected the top 50, 100, 150, 200, 250, and 300 DNMs that were most likely to be functional in patients with ASD based on PHRED-scaled scores and obtained corresponding thresholds to make predictions in unaffected siblings. We found that many methods yielded *P* > 0.05 and OR > 1.05 under the most relaxed condition (top 300). Under a more rigorous condition, some methods exhibited higher OR values and lower *P* values; CADD achieved the highest OR value and the lowest *P* value (OR = 1.5, *P* = 0.002, threshold = 21.6241), followed by CDTS (OR = 1.21, *P* = 0.0493, threshold = 26.8855). In summary, these results suggest that CADD and CDTS have better prediction performance for functional DNM.

### Different methods showed different resolutions

**Supplementary Figure S6:**
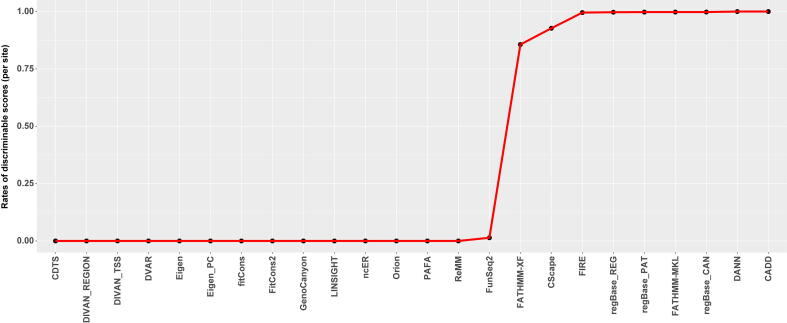


Theoretically, a perfect computational method should assign different prediction scores to different variants at the same position. Here, we calculated the rates of discriminable prediction scores among 24 computational methods for the same position across the whole genome, and noted that only nine methods, including regBase_REG [31], regBase_CAN [31], and regBase_PAT, showed discriminability at base-wise resolution for most sites in the whole genome (Figure S6). Additionally, for computational methods without discriminability at the base-wise resolution, we calculated the physical distances of surrounding DNA sites that showed the same prediction scores. We also determined the cumulative sum of proportions of different physical distances from 1 to the largest value until it was no smaller than 0.9, and then selected the last physical distances as the resolution. We found that most prediction scores of DNA sites differed with 1-bp site around them (Figure S7).

## Discussion

In recent years, it has been widely accepted that non-coding variants play important roles in human diseases [2], [3], [4], [5], [6], [7], [8], [9]. Many computational methods for evaluating the function and pathogenicity of non-coding variants have been developed for clinicians and geneticists to help them identify functional or pathogenic non-coding variants. Given that computational methods for non-coding variants have adopted various algorithms and training data based on different evolutionary constraints, epigenomics, and sequence features, their performance differs under differing conditions. However, it is difficult to choose an optimal method because of the lack of knowledge about the performance of the methods under different conditions. Selecting an optimal method can effectively aid in the prioritization of functional variants and candidate genes, thus increasing the demand for assessment of different computational methods under various conditions. In this work, we assessed 12 performance metrics of 24 computational methods based on four non-coding independent benchmark datasets.

Although multiple studies [35], [36], [37] have compared computational prediction methods for non-coding variants, our study differs from these studies for the following reasons. (1) Our benchmark data are more comprehensive and stricter. We constructed four benchmark datasets representing different genomic contexts and simulated realistic situations, such as positive and negative variants from the common regulatory variants from curated eQTL data with matched genomic features. (2) Our evaluation metrics are more comprehensive. We not only selected some classic metrics but also adopted hser-AUROC and hspr-AUROC data to serve some users who need to prioritize variants with high sensitivity or specificity. (3) To the best of our knowledge, this is the first study to assess the performance of existing methods for non-coding DNMs based on OR values.

Based on the correlation analysis of 24 computational methods, the predictive concordances among the 24 computational methods in rare somatic variants from COSMIC were higher than in the other three datasets. This may be because somatic variants result from replication errors and DNA damage [50]. Hence, somatic variants may have some similar features that germline variants do not, but most variants in the other three datasets are germline variants. Additionally, an ensemble learning method named regBase_CAN [31] in the prediction of common regulatory variants and disease-associated common variants was negatively correlated with many methods. Of note, most of these methods with a negative correlation with regBase_CAN were incorporated into regBase_CAN. Compared to other methods, regBase includes three methods designed for different purposes, and regBase_CAN is a method designed to predict the effects of somatic variants based on a somatic variant training dataset [31]. Thus, parameters in regBase_CAN may lead to inconsistent prediction results for common variants with other methods.

**Figure 2:**
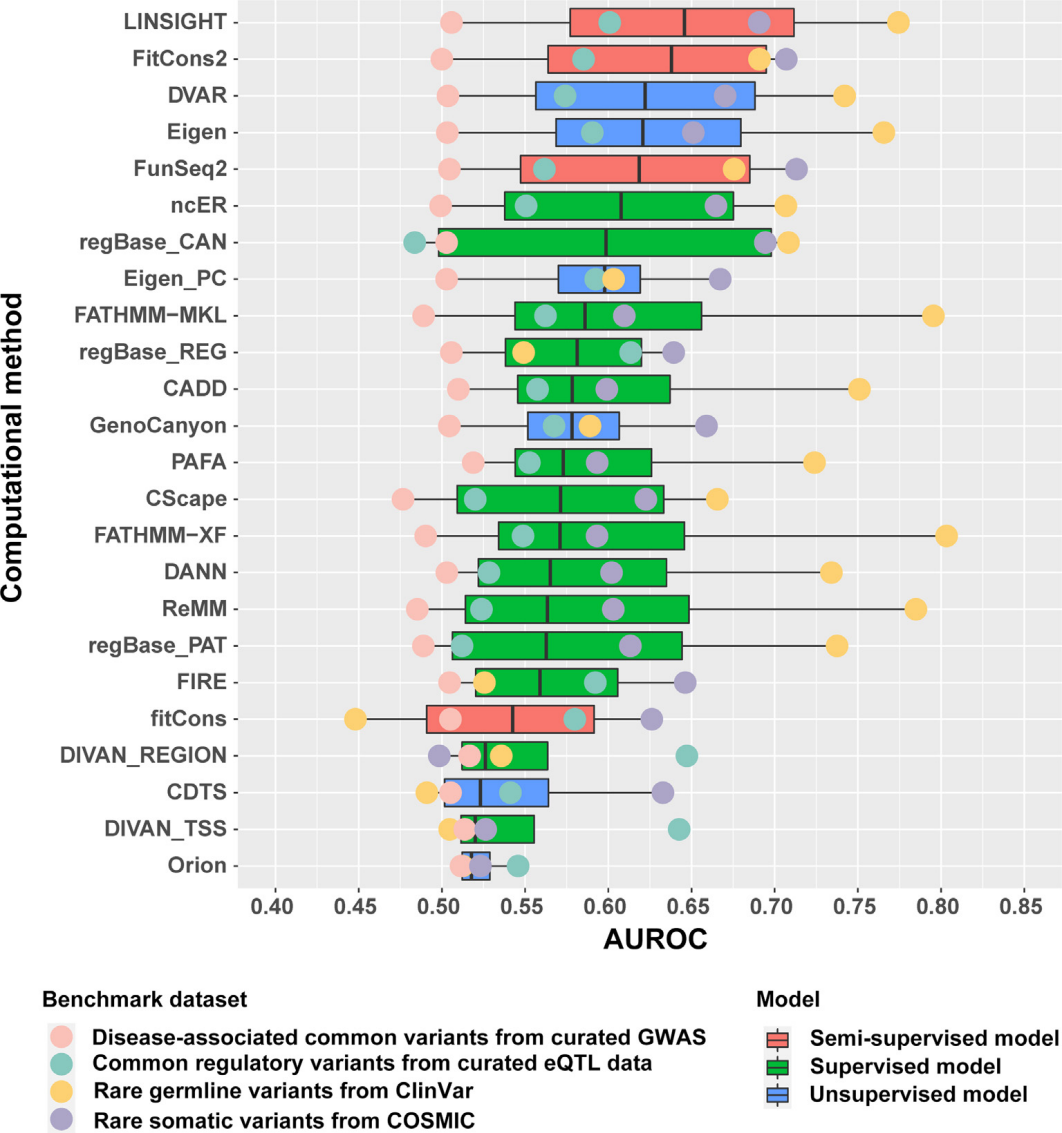
**Overall AUROC of four benchmark datasets** Distributions of AUROC values for 24 methods are shown in a boxplot. Differently colored balls represent different benchmark datasets. Differently colored boxes represent different models. AUROC, area under the receiver operating characteristic curve.

Based on our results, we clustered the 24 methods into three groups based on their computational models (supervised, unsupervised, and semi-supervised models), and preliminarily found that ncER (supervised model), DVAR (unsupervised model), and LINSIGH (semi-supervised model) [27] are the representative methods of the aforementioned three groups with the highest median of AUROC values based on four benchmark datasets (Figure 2). Additionally, we noted that computational methods showed different prediction efficiencies under different conditions (Figure 2). For example, FATHMM-XF was the best method for rare germline variants from ClinVar (AUROC = 0.8033) but performed poorly for rare somatic variants from COSMIC (AUROC = 0.5933). Although the performance of the computational methods varied for the four different benchmark datasets, the best performance was recorded for rare germline variants from ClinVar. These results are consistent with a previous study [35] and might be attributed to the following reasons. First, most computational methods selected more germline than somatic variants, which may have different genomic features; this selection bias in training data may improve performance in rare germline variant dataset from ClinVar. Second, it is well known that genetic variation in many complex quantitative traits results from the joint small effects of multiple variants [51], [52], and non-coding variants often have a weak impact on complex traits [53]. The stronger functional effects of germline variants in the ClinVar database made it easier to distinguish functional variants for these computational methods. Given that the contribution of single eQTL and GWAS SNV to heritability is small, functional prediction of these SNVs remains an enormous challenge.

In addition, we found that methods based on supervised models performed better than those based on unsupervised and semi-supervised models in rare germline variants from ClinVar. This may be explained by the selection of training data, as supervised learning demands representative and correctly labeled training data [54], and many methods based on supervised models select high-quality germline variant data from the Human Gene Mutation Database (HGMD) [55] and ClinVar database as training data. Thus, many methods based on supervised models performed better with rare germline variants from ClinVar. Furthermore, methods based on semi-supervised models performed better than unsupervised and supervised models in rare somatic variants from COSMIC. This may be because semi-supervised models select labeled and unlabeled data with stronger and weaker functional effects, respectively, as their training data. In contrast, the supervised and unsupervised models select labeled and unlabeled data, respectively, as their training data [54].

According to the performance measurement strategy, we divided the 24 methods into three groups (I, II, and III) based on the rank of their AUROC values, and every group contained eight methods (Table S7). None of these methods performed well in all evaluations. This may be because different evaluations represent different aspects of method performance. Hence, appropriate methods should be selected based on different requirements. In addition, the AUROC is not affected by different cutoff values and does not vary significantly with different ratios of positive and negative variants in benchmark data; thus, we selected the AUROC as our major measure.

It is well known that non-coding DNMs play important roles in neurodevelopmental disorders, such as ASD [46], [47], [48]; however, there is no authoritative database for validated pathogenic DNMs. To assess the prediction performance of the 24 methods for non-coding DNMs, we downloaded non-coding DNMs from patients with ASD and unaffected siblings from our previous study [49]. Although the pathogenicity of these DNMs is unclear, the number of pathogenic DNMs from patients with ASD should be more than unaffected siblings. Hence, we selected OR to assess the performance of these methods. In addition, we tried our best to collect 57 experimentally validated non-coding transcriptional-regulation-disruption DNMs from ASD probands and 50 nearest non-coding non-pathogenic DNMs in the siblings of ASD patients as our testing dataset to further assess the performance of 24 methods for DNMs. We noted that DVAR, regBase_CAN, and FitCons2 performed better with an AUROC > 0.77 (Table S8). Based on these results, we think it is still a challenge to make an accurate prediction for DNMs.

In this study, we noted that three of 24 compared methods were ensemble prediction models and found that the performances of the three methods (regBase_REG, regBase_PAT, and regBase_CAN) were moderate compared to other methods. In addition, we selected the top 10 methods of each benchmark dataset based on the sum of sensitivity and specificity to evaluate whether combined prediction would improve performance. If a variant was predicted as positive by more than half of the methods, it was considered positive. Finally, we assessed the performance of this combined prediction based on the accuracy and MCC, and found that combined prediction did not further improve performance. This indicates that it is still challenging to improve prediction performance for non-coding variants based on existing ensemble models. Hence, we think that more attention should be paid to improving the quality of training data and models to get better prediction performance for non-coding variants.

This study had some limitations. First, there was some potential circularity between the testing and training data of the computational prediction methods [56]. To eliminate potential circularity, we selected testing data that were recorded after 2019 and, as much as possible, removed variants that overlapped with publicly available training data when comparing methods. Given that some methods only provide the source and version without including the exact variants of the training data, a small amount of the benchmark data may still be the same as the training data in the methods. Hence, we suggest that scientists who develop new methods should publish their original training and testing data. Second, although the testing data downloaded from the ClinVar, COSMIC, the Genotype-Tissue Expression project (GTEx portal) [41], and GWAS catalog [57] databases have been widely used to develop computational methods and assess their performance, relatively little is known about the functional consequences of variations in the non-coding regions of the genome, and most variants in benchmark datasets were not experimentally validated; as such, incorrectly labeled data may have been included in our benchmark data. Therefore, we strongly recommend that scientists select experimentally validated or high-confidence training data to develop new methods in future studies.

Taken together, our findings suggest that existing computational methods show acceptable performance only for germline variants and that their predictive ability must be improved for different types of non-coding variants. We strongly recommend that more attention should be paid to the quality of learning data in future software development work. For example, methods should use various training data and genomic features to avoid selection bias. Our findings will serve as a useful guide for clinicians and researchers in choosing appropriate methods for non-coding variant prediction, leading to the development of new methods.

## Materials and methods

### Computational methods and prediction score processing

We compared 24 computational methods that provide precomputed prediction scores for the whole human genome. We included 14 methods based on supervised models, six based on unsupervised models, and four based on semi-supervised models (Table 1). The genomic positions of all precomputed scores were based on GRCh37/hg19. For standardization, all precomputed scores recorded by interval-level values were transformed into base-wise positions, and each base-wise position was assigned the same score. In addition, these raw scores were transformed into PHRED-scaled scores [−10 × log_10_ (rank/total)] according to the genome-wide distribution of scores for approximately 9 × 10^9^ potential SNVs, which is the set of all three non-reference alleles at each position of the reference assembly. PHRED-scaled scores provide a comparable unit to unify the estimation standard for assessment. For instance, if a raw score in the top 10% of all possible reference genomic SNVs, it was represented as a PHRED-scaled score of ≥ 10, and a raw score in the top 1‰ was represented as a score of ≥ 30. We calculated the mean of the precomputed base-level whole-genome DIsease-specific Variant ANnotation (DIVAN) [17] scores across 45 diseases for both region-matched and transcription start site (TSS)-matched criteria, and then transformed them into a PHRED-scaled score. Other raw and PHRED-scaled scores for all methods were downloaded from a previous study [31] except for DIVAN_TSS [17] and DIVAN_REGION [17].

### Benchmark datasets of non-coding variants

To evaluate the performance of the 24 methods, it was essential to construct an independent test of datasets in which variants overlapping with the training data were removed from the compared methods as much as possible. Four independent benchmark datasets of non-coding variants were used to assess the performance of the 24 computational methods, including (1) rare germline variants from ClinVar, (2) rare somatic variants from COSMIC, (3) common regulatory variants from curated eQTL data, and (4) disease-associated common variants from curated GWAS. Both positive and negative non-coding variants were included in each benchmark dataset (Table 2, Table S1). We adopted the following strategies to reduce overlap between testing benchmark data and training data for further analysis. First, as all training datasets were published before 2019, we selected variants recorded in public databases [38], [39] after 2019 to reduce overlap. Second, we comprehensively collected public training data on existing methods and removed overlap between benchmark data and available training data of the computational methods.

The first benchmark dataset (rare germline variants from ClinVar) was downloaded from the ClinVar database. According to the American College of Medical Genetics and Genomics guidelines [58], the variants were classified as ‘pathogenic’, ‘likely pathogenic’, ‘benign’, ‘likely benign’, and ‘uncertain significance’ in the ClinVar database. Furthermore, the ClinVar database contains interpretations of allele origins, and records in ClinVar with ORIGIN = 1 indicate that these variants are germline variants. To improve the accuracy of the benchmark dataset and eliminate overlap between testing benchmark data and training data used in the 24 computational methods, we selected all ‘pathogenic’, ‘likely pathogenic’, and ‘benign’ non-coding germline variants deposited in the ClinVar database after January 2, 2019, as testing data. And ‘pathogenic’ and ‘likely pathogenic’ non-coding germline variants are regarded as positive variants, and ‘benign’ non-coding germline variants are regarded as negative variants. Furthermore, we determined the AFs of these variants based on the gnomAD database, and noticed that (1) over 80% of ‘pathogenic’ and ‘likely pathogenic’ variants were not observed, (2) over 98% of ‘pathogenic’ and ‘likely pathogenic’ variants had AF < 0.1%, (3) over 99% of ‘benign’ variants were observed, and (4) over 98% of ‘benign’ variants had AF ≥ 0.1%. Based on the AFs of these variants, we regarded all ‘pathogenic’ and ‘likely pathogenic’ variants as rare variants with AF < 0.1%. Finally, we only selected all ‘pathogenic’, ‘likely pathogenic’, and ‘benign’ variants with AF < 0.1% (515 and 1850) as our testing data.

The second benchmark dataset (rare somatic variants from COSMIC) was downloaded from the COSMIC database. As most deleterious non-coding somatic variants are unknown and one criterion for identifying cancer driver variants is to examine their mutational recurrence across multiple samples [59], non-coding somatic variants from the COSMIC database after March 19, 2019 were divided into positive and negative variants, respectively, according to the recurrence of the variants. To increase the reliability of these variants, we also ensured that our positive variants are located on risk genes collected from the Cornell Non-coding Cancer driver Database (CNCDatabase) [40], whereas negative variants are not. A total of 2346 and 648,471 variants were categorized as positive and negative variants, respectively, when the threshold value of recurrence was equal to 2, and 84% of positive variants and 92% of negative variants had AF < 0.1% based on the gnomAD database. It is widely accepted that most somatic variants observed in the cancer genome are rare [60], and thus we only selected variants with AF < 0.1% (1966 and 597,222) as our final testing data.

It is well known that non-coding variants influence phenotypes mainly through regulating gene expression levels. Hence, we selected regulatory variants with minor allele frequency (MAF) > 5% as our third benchmark dataset (common regulatory variants from curated eQTL data) to assess the 24 methods. Here, we integrated three independent eQTL test datasets from two studies [18], [31] and removed eight variants labeled differently in both studies as our testing data. The positive dataset included (1) high-confidence eQTL single-nucleotide polymorphisms (SNPs) from the GTEx portal database and (2) multi-tissue eQTL SNP fine-mapping data from the GTEx portal database and Brown’s study [42]. The negative dataset was randomly sampled by vSampler [61] based on 1000 Genomes Project phase3 (1000G P3) [43], and negative variants were matched with positive variants based on the information of MAF, distance to the nearest transcription start site, gene density, and the number of variants in LD (Table S9). Notably, all positive and negative variants are non-coding, with MAF > 5% based on 1000G P3. We also referred to the criteria of test sets from Li’s study [36]. We only included paired positive and negative variants beyond 1 kb from each other as our final testing data to prevent physically proximate variants from confounding.

The fourth benchmark dataset (disease-associated common variants from curated GWAS) was downloaded from the CAUSALdb database [44] and 1000 Genomes Project [43]. We only selected non-coding SNVs in the intersection set of credible sets defined by three fine-mapping tools, including probabilistic annotation integrator (PAINTOR) [62], caviar bayes factor (CAVIARBF) [63], and FINEMAP [64] with MAF > 5% based on the 1000 Genomes Project as positive variants and corresponding non-coding SNVs in the same LD blocks with R^2^ > 0.2 from the 1000 Genomes Project with MAF > 5% as negative variants. Overlapping variants between positive and negative data as well as positive variants without corresponding negative variants were excluded from the analysis.

### Correlation analysis

Spearman rank correlation analysis was used to evaluate the relationships among the 24 compared computational methods based on the four non-coding benchmark datasets described above. Specifically, Spearman rank correlation coefficients were calculated between any two computational methods for each benchmark dataset, in which variants with missing values for a method were excluded, and the results of correlation analyses were visualized in the form of heatmaps. In addition, for each benchmark dataset, we performed correlation analysis based on the positive and negative variant datasets.

### Metrics for performance evaluation

The performances of the 24 computational methods were assessed based on the following 12 criteria: (1) the positive predictive value (PPV), the proportion of positive results in the computational methods that are positive under the benchmark dataset; (2) the negative predictive value (NPV), the proportion of negative results in computational methods that are negative under the benchmark dataset; (3) the false-negative rate (FNR), which is calculated as the ratio of the number of positive events wrongly categorized as negative by the computational method to the total number of actual positive events under the benchmark dataset; (4) the sensitivity (or true-positive rate; TPR), which measures the proportion of actual positives under the benchmark dataset that are correctly identified as such by the computational method. The FNR and sensitivity are paired measures with a sum equal to 100%; (5) the false-positive rate (FPR), which is calculated as the ratio of the number of negative events wrongly categorized as positive by the computational method to the total number of actual negative events under the benchmark dataset; (6) the specificity (or true-negative rate; TNR), which measures the proportion of actual negatives under the benchmark dataset that are correctly identified as such by the computational method. The FPR and specificity are paired metrics with a sum equal to 100%; (7) the accuracy, which represents the proportion of positive and negative variants in the benchmark data that are correctly predicted as positive and negative variants, respectively; (8) the MCC, a correlation coefficient (ranging from −1 to 1) between the observed and predicted classifications (where 1 indicates a perfect prediction, 0 indicates no better than random prediction, and −1 indicates complete disagreement between the prediction and true classification); (9) the ROC curve, a graphical plot that illustrates the predictive ability of a computational method as its discrimination thresholds are varied; (10) the AUROC value, which ranges from 0 to 1 for each ROC curve, where a higher AUROC indicates better performance of the computational method; (11) the hser-AUROC value, which is the AUROC corresponding to high sensitivity (TPR > 95%); and (12) the hspr-AUROC value, which is the AUROC corresponding to high specificity (TNR > 95%). The hser-AUROC and hspr-AUROC values are evaluated to serve some users who require a distinction between positive variants with high sensitivity or specificity. Given that many computational methods do not offer recommended cutoff values, all metrics described above were calculated based on the best thresholds corresponding to the best sum of sensitivity and specificity. In addition, the best thresholds, sensitivities, specificities, AUROC values, hspr-AUROC values, and hser-AUROC values were calculated using the ‘pROC’ package [65] based on PHRED-scaled scores.

### Non-coding DNMs from the Simons simplex collection

Non-coding DNMs identified in 1902 patients with ASD and 1902 unaffected siblings were downloaded from the Simons simplex collection [47], [66] (Table S1) and were previously cataloged in the Gene4Denovo database that we developed [49]. Comparison of the performance of computational methods for non-coding DNMs was based on PHRED-scaled scores. We compared the burden of functional non-coding variants predicted by the computational methods in the ASD and sibling groups under different cutoff values. To assess the performance of computational methods for DNMs, we calculated the OR, 95% confidence interval of the OR, and *P* value between ASD and unaffected siblings using the two-sided Poisson’s ratio test.

### Experimentally validated non-coding DNMs from ASD

We collected experimentally validated non-coding transcriptional-regulation-disruption DNMs from ASD probands [48] and nearest non-coding non-pathogenic DNMs in the siblings of ASD patients [31] as our supplementary test dataset (Table S1) to further assess the performance of 24 methods for DNMs.

## Competing interests

The authors have declared no competing interests.

## CRediT authorship contribution statement

**Zheng Wang:** Conceptualization, Methodology, Validation, Formal analysis, Investigation, Data curation, Writing – original draft, Writing – review & editing. **Guihu Zhao:** Conceptualization, Methodology, Software, Data curation, Writing – original draft. **Bin Li:** Investigation. **Zhenghuan Fang:** Methodology. **Qian Chen:** Investigation. **Xiaomeng Wang:** Data curation. **Tengfei Luo:** Investigation. **Yijing Wang:** Investigation. **Qiao Zhou:** Investigation, Data curation. **Kuokuo Li:** Visualization. **Lu Xia:** Investigation. **Yi Zhang:** Investigation. **Xun Zhou:** Investigation, Data curation, Visualization. **Hongxu Pan:** Investigation, Data curation, Visualization. **Yuwen Zhao:** Investigation, Data curation, Visualization. **Yige Wang:** Investigation, Data curation, Visualization. **Lin Wang:** Data curation. **Jifeng Guo:** Resources, Supervision, Project administration. **Beisha Tang:** Conceptualization, Resources, Writing – review & editing. **Kun Xia:** Resources, Supervision, Project administration. **Jinchen Li:** Conceptualization, Resources, Writing – review & editing, Supervision, Project administration, Funding acquisition. All authors have read and approved the final manuscript.

## Figures and Tables

**Supplementary Figure S7:**