# Supplementary material for: Performance Comparison of Computational Methods for the Prediction of the Function and Pathogenicity of Non-coding Variants
Source: Genomics Proteomics Bioinformatics. 2022 Mar 8;21(3):649–61. doi: 10.1016/j.gpb.2022.02.002 (PMC10787016; doi:10.1016/j.gpb.2022.02.002)
Supplement: Supplementary Table S2 [file mmc2.docx]

**Table S2 Performance evaluation based on rare germline variants from ClinVar without ‘likely pathogenic’ variants**

| Methods | Missing rate (%) | Best-threshold | PPV (%) | NPV (%) | FNR (%) | Sensitivity (%) | FPR (%) | Specificity (%) | Accuracy (%) | MCC | AUC | hspr-AUC | hser-AUC | Prediction model |
| --- | --- | --- | --- | --- | --- | --- | --- | --- | --- | --- | --- | --- | --- | --- |
| CADD | 0.00 | 9.9287 | 30.31 | 91.97 | 33.82 | 66.18 | 28.22 | 71.78 | 70.91 | 0.2908 | 0.7292 | 0.5559 | 0.5237 | SM |
| CScape | 8.98 | 30.6132 | 27.14 | 88.89 | 52.60 | 47.40 | 23.22 | 76.78 | 72.24 | 0.1969 | 0.6434 | 0.5317 | 0.5302 | SM |
| DANN | 0.00 | 9.5376 | 34.12 | 89.92 | 49.56 | 50.44 | 18.05 | 81.95 | 77.02 | 0.2790 | 0.7124 | 0.5795 | 0.5228 | SM |
| DIVAN_REGION | 0.00 | 2.7014 | 17.58 | 88.06 | **26.24** | **73.76** | 64.11 | 35.89 | 41.81 | 0.0738 | 0.5419 | 0.5112 | 0.5074 | SM |
| DIVAN_TSS | 0.00 | 3.8883 | 17.36 | 87.23 | 30.61 | 69.39 | 61.24 | 38.76 | 43.55 | 0.0611 | 0.5117 | 0.5010 | 0.5042 | SM |
| FATHMM-MKL | 0.00 | 12.1444 | 38.38 | **92.81** | 33.53 | 66.47 | 19.78 | 80.22 | 78.07 | 0.3816 | **0.7866** | **0.6364** | **0.5369** | SM |
| FATHMM-XF | 8.98 | 26.0395 | **49.61** | **92.84** | 37.34 | 62.66 | **11.61** | **88.39** | **84.42** | **0.4656** | **0.7918** | **0.6914** | 0.5099 | SM |
| FIRE | 0.00 | 10.2942 | 21.07 | 87.26 | 53.06 | 46.94 | 32.59 | 67.41 | 64.20 | 0.1094 | 0.5499 | NA | 0.5079 | SM |
| ncER | 0.27 | 13.7907 | 29.86 | 91.49 | 36.26 | 63.74 | 27.75 | 72.25 | 70.92 | 0.2772 | 0.7014 | 0.5249 | 0.5118 | SM |
| PAFA | 7.30 | 1.1395 | 27.71 | 92.71 | 28.34 | 71.66 | 34.15 | 65.85 | 66.75 | 0.2768 | 0.6951 | 0.5171 | NA | SM |
| regBase_CAN | 0.00 | 10.2935 | 29.71 | 92.30 | 31.49 | 68.51 | 30.05 | 69.95 | 69.72 | 0.2909 | 0.7066 | 0.5236 | 0.5036 | SM |
| regBase_PAT | 0.00 | 7.3824 | 26.06 | 92.18 | 28.57 | 71.43 | 37.57 | 62.43 | 63.84 | 0.2485 | 0.7255 | 0.5786 | **0.5471** | SM |
| regBase_REG | 0.00 | 22.6985 | 21.44 | 85.83 | 72.30 | 27.70 | 18.81 | 81.19 | 72.82 | 0.0804 | 0.5473 | NA | NA | SM |
| ReMM | 0.00 | 15.7836 | **43.64** | 91.39 | 44.02 | 55.98 | 13.41 | 86.59 | **81.81** | **0.3861** | **0.7638** | 0.6007 | 0.5298 | SM |
| CDTS | 8.62 | 9.6805 | 16.36 | 85.10 | 65.58 | 34.42 | 31.96 | 68.04 | 62.87 | 0.0190 | 0.4579 | NA | NA | UM |
| DVAR | 0.00 | 15.0531 | 40.36 | 91.62 | 41.40 | 58.60 | 16.05 | 83.95 | 79.98 | 0.3689 | 0.7283 | 0.5387 | 0.5161 | UM |
| Eigen | 9.35 | 13.9948 | 30.66 | **92.86** | 30.69 | 69.31 | 28.19 | 71.81 | 71.43 | 0.3110 | 0.7552 | 0.5351 | **0.5425** | UM |
| Eigen_PC | 9.35 | 8.7340 | 20.83 | 91.74 | **24.09** | **75.91** | 51.87 | 48.13 | 52.36 | 0.1739 | 0.5992 | NA | 0.5356 | UM |
| GenoCanyon | 0.00 | 16.3293 | 30.65 | 86.83 | 72.30 | 27.70 | 11.62 | 88.38 | 78.89 | 0.1676 | 0.5699 | 0.5459 | NA | UM |
| Orion | 14.41 | 17.3378 | 23.08 | 86.11 | 86.91 | 13.09 | **7.49** | **92.51** | 80.87 | 0.0717 | 0.5159 | 0.5137 | NA | UM |
| fitCons | 7.39 | 6.0945 | 15.85 | 85.54 | **26.11** | **73.89** | 71.75 | 28.25 | 35.30 | 0.0172 | 0.4584 | NA | 0.5012 | SSM |
| FitCons2 | 7.30 | 17.3066 | 32.72 | 89.98 | 49.36 | 50.64 | 19.02 | 80.98 | 76.29 | 0.2679 | 0.6777 | 0.5880 | 0.5005 | SSM |
| FunSeq2 | 1.50 | 11.6588 | 25.90 | 90.07 | 40.41 | 59.59 | 31.74 | 68.26 | 66.90 | 0.2109 | 0.6727 | 0.5385 | 0.5262 | SSM |
| LINSIGHT | 1.73 | 16.9706 | **49.87** | 91.67 | 43.92 | 56.08 | **10.45** | **89.55** | **84.32** | **0.4354** | 0.7609 | **0.6202** | 0.5000 | SSM |

*Note*: Best-threshold, the threshold corresponding to the best sum of sensitivity and specificity; PPV, positive predictive value; NPV, negative predictive value; FPR, false positive rate; FNR, false negative rate; MCC, mathew correlation coefficient; AUC, area under the curve; hspr-AUC, high-specificity regional area under the curve; hser-AUC, high-sensitivity regional area under the curve; NA, not available; SM, supervised model; UM, unsupervised model; SSM, semi-supervised model. Top three methods of every measure are represented by bold text.
