# Supplementary material for: Performance Comparison of Computational Methods for the Prediction of the Function and Pathogenicity of Non-coding Variants
Source: Genomics Proteomics Bioinformatics. 2022 Mar 8;21(3):649–61. doi: 10.1016/j.gpb.2022.02.002 (PMC10787016; doi:10.1016/j.gpb.2022.02.002)
Supplement: Supplementary Table S3 [file mmc3.docx]

**Table S3 Performance evaluation based on rare somatic variants from COSMIC**

| Methods | Missing rate (%) | Best-threshold | PPV (%) | NPV (%) | FNR (%) | Sensitivity (%) | FPR (%) | Specificity (%) | Accuracy (%) | MCC | AUC | hspr-AUC | hser-AUC | Prediction model |
| --- | --- | --- | --- | --- | --- | --- | --- | --- | --- | --- | --- | --- | --- | --- |
| CADD | 0.00 | 6.7405 | 0.49 | 99.76 | 46.64 | 53.36 | 35.39 | 64.61 | 64.58 | 0.0215 | 0.5991 | 0.5082 | NA | SM |
| CScape | 7.32 | 9.1969 | 0.48 | 99.78 | 49.26 | 50.74 | **32.40** | **67.60** | **67.55** | 0.0216 | 0.6226 | 0.5262 | 0.5151 | SM |
| DANN | 0.00 | 7.6538 | 0.52 | 99.74 | 58.60 | 41.40 | **26.03** | **73.97** | **73.87** | 0.0200 | 0.6020 | 0.5289 | 0.5056 | SM |
| DIVAN_REGION | 0.00 | 1.5314 | 0.35 | 99.78 | **12.92** | **87.08** | 80.54 | 19.46 | 19.68 | 0.0094 | 0.4984 | NA | 0.5066 | SM |
| DIVAN_TSS | 0.00 | 2.0320 | 0.38 | 99.83 | **12.46** | **87.54** | 76.31 | 23.69 | 23.90 | 0.0151 | 0.5263 | NA | 0.5087 | SM |
| FATHMM-MKL | 0.02 | 7.6906 | 0.49 | 99.76 | 46.80 | 53.20 | 35.57 | 64.43 | 64.39 | 0.0211 | 0.6096 | 0.5185 | 0.5013 | SM |
| FATHMM-XF | 7.32 | 10.7800 | 0.48 | 99.79 | 44.85 | 55.15 | 34.97 | 65.03 | 65.00 | 0.0234 | 0.5933 | 0.5196 | NA | SM |
| FIRE | 0.00 | 4.9090 | 0.46 | **99.87** | 15.51 | 84.49 | 60.16 | 39.84 | 39.99 | 0.0284 | 0.6462 | 0.5072 | 0.5897 | SM |
| ncER | 0.78 | 6.8488 | 0.51 | 99.83 | 28.30 | 71.70 | 45.83 | 54.17 | 54.22 | 0.0297 | 0.6647 | 0.5135 | 0.5448 | SM |
| PAFA | 2.82 | 6.4113 | 0.48 | 99.78 | 37.26 | 62.74 | 43.53 | 56.47 | 56.49 | 0.0223 | 0.5933 | 0.5196 | NA | SM |
| regBase_CAN | 0.00 | 7.3988 | **0.58** | 99.84 | 30.01 | 69.99 | 39.69 | 60.31 | 60.34 | **0.0354** | **0.6944** | 0.5132 | 0.5707 | SM |
| regBase_PAT | 0.00 | 6.0064 | 0.49 | 99.76 | 47.76 | 52.24 | 35.14 | 64.86 | 64.82 | 0.0205 | 0.6133 | 0.5225 | 0.5033 | SM |
| regBase_REG | 0.00 | 9.2831 | 0.48 | 99.78 | 39.57 | 60.43 | 40.82 | 59.18 | 59.19 | 0.0228 | 0.6393 | 0.5157 | 0.5389 | SM |
| ReMM | 0.00 | 8.0817 | 0.49 | 99.76 | 49.24 | 50.76 | 33.96 | 66.04 | 65.99 | 0.0203 | 0.6030 | 0.5192 | 0.5182 | SM |
| CDTS | 14.24 | 7.7832 | 0.46 | 99.81 | 44.52 | 55.48 | 34.06 | 65.94 | 65.91 | 0.0241 | 0.6328 | 0.5115 | 0.5084 | UM |
| DVAR | 0.00 | 6.9717 | 0.51 | 99.84 | 25.38 | 74.62 | 47.76 | 52.24 | 52.31 | 0.0307 | 0.6702 | 0.5111 | 0.5645 | UM |
| Eigen | 5.96 | 9.0256 | 0.50 | 99.82 | 37.62 | 62.38 | 37.69 | 62.31 | 62.31 | 0.0281 | 0.6511 | 0.5232 | 0.5060 | UM |
| Eigen_PC | 5.96 | 7.2917 | 0.49 | 99.84 | 28.84 | 71.16 | 44.63 | 55.37 | 55.42 | 0.0294 | 0.6673 | 0.5243 | 0.5133 | UM |
| GenoCanyon | 0.00 | 7.8766 | **0.56** | 99.78 | 46.29 | 53.71 | **31.57** | **68.43** | **68.39** | 0.0272 | 0.6590 | **0.5368** | 0.5374 | UM |
| Orion | 27.71 | 7.9485 | 0.46 | 99.65 | 46.08 | 53.92 | 46.82 | 53.18 | 53.18 | 0.0090 | 0.5232 | 0.5001 | NA | UM |
| fitCons | 2.93 | 12.0551 | 0.55 | 99.79 | 41.70 | 58.30 | 35.27 | 64.73 | 64.71 | 0.0277 | 0.6262 | **0.5331** | NA | SSM |
| FitCons2 | 2.82 | 11.3451 | **0.59** | 99.84 | 28.46 | 71.54 | 40.55 | 59.45 | 59.49 | **0.0364** | **0.7069** | 0.5325 | **0.5942** | SSM |
| FunSeq2 | 3.97 | 4.5040 | 0.47 | **99.90** | 17.02 | 82.98 | 51.70 | 48.30 | 48.41 | **0.0340** | **0.7131** | **0.5487** | **0.6205** | SSM |
| LINSIGHT | 4.69 | 6.1970 | 0.46 | **99.90** | **14.83** | **85.17** | 55.69 | 44.31 | 44.43 | 0.0325 | 0.6907 | 0.5163 | **0.5967** | SSM |

*Note*: COSMIC, catalogue of somatic mutations in cancer; Best-threshold, the threshold corresponding to the best sum of sensitivity and specificity; PPV, positive predictive value; NPV, negative predictive value; FPR, false positive rate; FNR, false negative rate; MCC, mathew correlation coefficient; AUC, area under the curve; hspr-AUC, high-specificity regional area under the curve; hser-AUC, high-sensitivity regional area under the curve; NA, not available; SM, supervised model; UM, unsupervised model; SSM, semi-supervised model. Top three methods of every measure are represented by bold text.
