# Supplementary material for: Performance Comparison of Computational Methods for the Prediction of the Function and Pathogenicity of Non-coding Variants
Source: Genomics Proteomics Bioinformatics. 2022 Mar 8;21(3):649–61. doi: 10.1016/j.gpb.2022.02.002 (PMC10787016; doi:10.1016/j.gpb.2022.02.002)
Supplement: Supplementary Table S4 [file mmc4.docx]

**Table S4 Performance evaluation based on common regulatory variants from curated eQTL data**

| Methods | Missing rate (%) | Best-threshold | PPV (%) | NPV (%) | FNR (%) | Sensitivity (%) | FPR (%) | Specificity (%) | Accuracy (%) | MCC | AUC | hspr-AUC | hser-AUC | Prediction model |
| --- | --- | --- | --- | --- | --- | --- | --- | --- | --- | --- | --- | --- | --- | --- |
| CADD | 0.00 | 1.8401 | 53.57 | 55.84 | 33.52 | 66.48 | 57.61 | 42.39 | 54.43 | 0.0914 | 0.5575 | 0.5021 | 0.5102 | SM |
| CScape | 1.51 | 3.1122 | 55.12 | 50.85 | 72.11 | 27.89 | **23.33** | **76.67** | 51.95 | 0.0522 | 0.5201 | 0.5058 | 0.5022 | SM |
| DANN | 0.00 | 0.7594 | 51.72 | 56.43 | 18.38 | 81.62 | 76.19 | 23.81 | 52.71 | 0.0665 | 0.5283 | NA | 0.5142 | SM |
| DIVAN_REGION | 0.00 | 2.4827 | **58.20** | **69.58** | **17.95** | **82.05** | 58.93 | 41.07 | **61.56** | **0.2534** | **0.6472** | **0.5104** | **0.5723** | SM |
| DIVAN_TSS | 0.00 | 2.9377 | 58.15 | **69.30** | **18.24** | **81.76** | 58.84 | 41.16 | **61.46** | **0.2508** | **0.6426** | 0.5066 | **0.5714** | SM |
| FATHMM-MKL | 0.00 | 1.5323 | 54.30 | 55.93 | 37.06 | 62.94 | 52.96 | 47.04 | 54.99 | 0.1010 | 0.5623 | 0.5004 | 0.5152 | SM |
| FATHMM-XF | 1.51 | 1.5933 | 57.30 | 52.41 | 64.04 | 35.96 | **27.54** | **72.46** | 53.97 | 0.0905 | 0.5488 | **0.5138** | 0.5003 | SM |
| FIRE | 0.00 | 5.8832 | 56.37 | 58.16 | 36.70 | 63.30 | 48.99 | 51.01 | 57.15 | 0.1442 | 0.5922 | 0.5074 | 0.5179 | SM |
| ncER | 0.34 | 2.7862 | 54.02 | 54.63 | 41.93 | 58.07 | 49.47 | 50.53 | 54.30 | 0.0862 | 0.5506 | 0.5001 | 0.5088 | SM |
| PAFA | 0.00 | 1.6172 | 54.67 | 53.89 | 50.32 | 49.68 | 41.19 | 58.81 | 54.25 | 0.0853 | 0.5526 | 0.5054 | NA | SM |
| regBase_CAN | 0.00 | 1.6270 | 50.22 | 52.02 | **9.50** | **90.50** | 89.70 | 10.30 | 50.40 | 0.0134 | 0.4837 | NA | 0.5022 | SM |
| regBase_PAT | 0.00 | 2.9755 | 52.06 | 51.30 | 59.72 | 40.28 | 37.10 | 62.90 | 51.59 | 0.0327 | 0.5121 | NA | NA | SM |
| regBase_REG | 0.00 | 5.7329 | 57.49 | 57.87 | 41.10 | 58.90 | 43.55 | 56.45 | 57.67 | 0.1535 | **0.6134** | **0.5148** | **0.5262** | SM |
| ReMM | 0.00 | 5.4273 | 55.96 | 51.69 | 75.29 | 24.71 | **19.44** | **80.56** | 52.63 | 0.0635 | 0.5238 | 0.5057 | 0.5048 | SM |
| CDTS | 9.68 | 3.4039 | 55.45 | 51.14 | 44.83 | 55.17 | 48.57 | 51.43 | 53.38 | 0.0660 | 0.5412 | 0.5031 | 0.5059 | UM |
| DVAR | 0.00 | 4.6740 | 54.73 | 57.70 | 32.17 | 67.83 | 56.12 | 43.88 | 55.86 | 0.1207 | 0.5740 | 0.5023 | 0.5116 | UM |
| Eigen | 0.75 | 2.7022 | 57.24 | 57.42 | 39.86 | 60.14 | 45.53 | 54.47 | 57.33 | 0.1464 | 0.5904 | 0.5072 | 0.5143 | UM |
| Eigen_PC | 0.75 | 2.9487 | 56.83 | **59.09** | 33.15 | 66.85 | 51.46 | 48.54 | 57.75 | 0.1565 | 0.5924 | 0.5064 | 0.5173 | UM |
| GenoCanyon | 0.00 | 5.2397 | 54.40 | 55.35 | 40.29 | 59.71 | 50.06 | 49.94 | 54.83 | 0.0970 | 0.5674 | 0.5073 | 0.5160 | UM |
| Orion | 38.34 | 4.6326 | **60.30** | 47.42 | 29.32 | 70.68 | 63.76 | 36.24 | 56.15 | 0.0731 | 0.5458 | 0.5030 | 0.5124 | UM |
| fitCons | 0.02 | 8.6616 | **58.55** | 54.98 | 56.90 | 43.10 | 30.51 | 69.49 | 56.30 | 0.1305 | 0.5799 | NA | 0.5010 | SSM |
| FitCons2 | 0.00 | 6.8789 | 56.44 | 58.01 | 37.43 | 62.57 | 48.28 | 51.72 | 57.14 | 0.1437 | 0.5851 | NA | 0.5128 | SSM |
| FunSeq2 | 1.43 | 5.2371 | 54.77 | 54.05 | 41.95 | 58.05 | 49.28 | 50.72 | 54.44 | 0.0880 | 0.5616 | 0.5085 | 0.5056 | SSM |
| LINSIGHT | 1.51 | 4.7214 | 57.41 | 58.44 | 34.80 | 65.20 | 49.71 | 50.29 | **57.85** | **0.1567** | 0.6009 | 0.5089 | 0.5161 | SSM |

*Note*: eQTL, expression quantitative trait locus; Best-threshold, the threshold corresponding to the best sum of sensitivity and specificity; PPV, positive predictive value; NPV, negative predictive value; FPR, false positive rate; FNR, false negative rate; MCC, mathew correlation coefficient; AUC, area under the curve; hspr-AUC, high-specificity regional area under the curve; hser-AUC, high-sensitivity regional area under the curve; NA, not available; SM, supervised model; UM, unsupervised model; SSM, semi-supervised model. Top three methods of every measure are represented by bold text.
