# Supplementary material for: Performance Comparison of Computational Methods for the Prediction of the Function and Pathogenicity of Non-coding Variants
Source: Genomics Proteomics Bioinformatics. 2022 Mar 8;21(3):649–61. doi: 10.1016/j.gpb.2022.02.002 (PMC10787016; doi:10.1016/j.gpb.2022.02.002)
Supplement: Supplementary Table S5 [file mmc5.docx]

**Table S5 Performance evaluation based on disease-associated common variants from curated GWAS**

| Methods | Missing rate (%) | Best-threshold | PPV (%) | NPV (%) | FNR (%) | Sensitivity (%) | FPR (%) | Specificity (%) | Accuracy (%) | MCC | AUC | hspr-AUC | hser-AUC | Prediction model |
| --- | --- | --- | --- | --- | --- | --- | --- | --- | --- | --- | --- | --- | --- | --- |
| CADD | 0.00 | 1.3843 | 0.50 | 0.52 | 0.36 | 0.64 | 0.63 | 0.37 | **0.51** | 0.0182 | 0.5099 | 0.5002 | 0.5020 | SM |
| CScape | 0.00 | 27.2206 | **0.59** | 0.51 | 1.00 | 0.00 | **0.00** | **1.00** | **0.51** | 0.0059 | 0.4766 | NA | NA | SM |
| DANN | 0.00 | 0.2798 | 0.50 | **0.53** | **0.13** | **0.87** | 0.86 | 0.14 | 0.50 | 0.0187 | 0.5030 | NA | 0.5028 | SM |
| DIVAN_REGION | 0.00 | 2.0742 | 0.50 | 0.52 | 0.39 | 0.61 | 0.58 | 0.42 | **0.51** | **0.0259** | **0.5167** | **0.5014** | **0.5036** | SM |
| DIVAN_TSS | 0.00 | 1.8537 | 0.50 | 0.52 | 0.35 | 0.65 | 0.63 | 0.37 | **0.51** | 0.0216 | **0.5137** | 0.5007 | **0.5037** | SM |
| FATHMM-MKL | 0.00 | 10.4953 | 0.50 | 0.51 | 0.95 | 0.05 | 0.05 | 0.95 | **0.51** | 0.0043 | 0.4890 | 0.5005 | NA | SM |
| FATHMM-XF | 0.00 | 4.8704 | 0.49 | 0.51 | 0.77 | 0.23 | 0.23 | 0.77 | **0.51** | 0.0017 | 0.4903 | 0.5001 | NA | SM |
| FIRE | 0.00 | 8.0711 | 0.50 | 0.51 | 0.78 | 0.22 | 0.21 | 0.79 | **0.51** | 0.0104 | 0.5046 | 0.5009 | 0.5002 | SM |
| ncER | 0.00 | 11.3622 | **0.51** | 0.51 | 0.96 | 0.04 | 0.04 | 0.96 | **0.51** | 0.0091 | 0.4993 | **0.5010** | 0.5003 | SM |
| PAFA | 0.00 | 0.5155 | **0.51** | **0.53** | 0.44 | 0.56 | 0.52 | 0.48 | **0.52** | **0.0332** | **0.5188** | 0.5002 | 0.5023 | SM |
| regBase_CAN | 0.00 | 2.5935 | 0.49 | 0.52 | 0.22 | 0.78 | 0.77 | 0.23 | 0.50 | 0.0087 | 0.5029 | NA | 0.5007 | SM |
| regBase_PAT | 0.00 | 14.6167 | 0.50 | 0.51 | 0.99 | 0.01 | **0.01** | **0.99** | **0.51** | 0.0007 | 0.4889 | NA | NA | SM |
| regBase_REG | 0.00 | 3.0243 | 0.50 | 0.51 | 0.43 | 0.57 | 0.56 | 0.44 | 0.50 | 0.0113 | 0.5057 | **0.5010** | 0.5005 | SM |
| ReMM | 0.00 | 37.0900 | **0.75** | 0.51 | 1.00 | 0.00 | **0.00** | **1.00** | **0.51** | 0.0027 | 0.4853 | NA | NA | SM |
| CDTS | 0.17 | 0.8700 | 0.50 | 0.52 | **0.12** | **0.88** | 0.87 | 0.13 | 0.50 | 0.0147 | 0.5052 | 0.5003 | 0.5019 | UM |
| DVAR | 0.00 | 2.9796 | 0.50 | 0.51 | 0.45 | 0.55 | 0.55 | 0.45 | 0.50 | 0.0086 | 0.5036 | 0.5008 | NA | UM |
| Eigen | 0.00 | 2.7579 | 0.50 | 0.51 | 0.60 | 0.40 | 0.39 | 0.61 | **0.51** | 0.0115 | 0.5033 | 0.5009 | NA | UM |
| Eigen_PC | 0.00 | 2.3400 | 0.50 | 0.51 | 0.50 | 0.50 | 0.49 | 0.51 | 0.50 | 0.0076 | 0.5029 | 0.5004 | 0.5001 | UM |
| GenoCanyon | 0.00 | 2.6909 | 0.50 | 0.51 | 0.40 | 0.60 | 0.60 | 0.40 | 0.50 | 0.0086 | 0.5045 | 0.5002 | 0.5006 | UM |
| Orion | 0.55 | 3.3963 | 0.51 | **0.56** | **0.06** | **0.94** | 0.92 | 0.08 | **0.51** | **0.0358** | 0.5115 | 0.5007 | **0.5063** | UM |
| fitCons | 0.00 | 4.5007 | 0.50 | 0.51 | 0.58 | 0.42 | 0.42 | 0.58 | **0.51** | 0.0081 | 0.5052 | 0.5001 | 0.5006 | SSM |
| FitCons2 | 0.00 | 4.6549 | 0.49 | 0.51 | 0.59 | 0.41 | 0.41 | 0.59 | 0.50 | 0.0050 | 0.5000 | NA | 0.5000 | SSM |
| FunSeq2 | 0.00 | 1.1356 | 0.49 | 0.52 | 0.22 | 0.78 | 0.77 | 0.23 | 0.50 | 0.0120 | 0.5046 | 0.5005 | NA | SSM |
| LINSIGHT | 0.00 | 3.5183 | 0.50 | 0.51 | 0.53 | 0.47 | 0.46 | 0.54 | **0.51** | 0.0097 | 0.5058 | 0.5007 | 0.5006 | SSM |

*Note*: GWAS, genome-wide association studies. Best-threshold, the threshold corresponding to the best sum of sensitivity and specificity; PPV, positive predictive value; NPV, negative predictive value; FPR, false positive rate; FNR, false negative rate; MCC, mathew correlation coefficient; AUC, area under the curve; hspr-AUC, high-specificity regional area under the curve; hser-AUC, high-sensitivity regional area under the curve; NA, not available; SM, supervised model; UM, unsupervised model; SSM, semi-supervised model. Top three methods of every measure are represented by bold text.
