# Supplementary material for: Performance Comparison of Computational Methods for the Prediction of the Function and Pathogenicity of Non-coding Variants
Source: Genomics Proteomics Bioinformatics. 2022 Mar 8;21(3):649–61. doi: 10.1016/j.gpb.2022.02.002 (PMC10787016; doi:10.1016/j.gpb.2022.02.002)
Supplement: Supplementary Table S6 [file mmc6.docx]

**Table S6 Performance evaluation based on *de novo* mutations dataset**

| Method | Threshold | ASD | Sibling | OR | %95CI | %95CI | *P* value | Group |
| --- | --- | --- | --- | --- | --- | --- | --- | --- |
| CADD | 10.0000 | 9,498 | 9,232 | 1.0288 | 0.9997 | 1.0588 | 0.0528 | Pathogenic threshold = 10 |
| CDTS | 10.0000 | 10,861 | 10,533 | 1.0311 | 1.0038 | 1.0593 | 0.0254 | Pathogenic threshold = 10 |
| CScape | 10.0000 | 6,792 | 6,594 | 1.0300 | 0.9956 | 1.0657 | 0.0886 | Pathogenic threshold = 10 |
| DANN | 10.0000 | 9,478 | 9,005 | 1.0525 | 1.0225 | 1.0834 | 0.0005 | Pathogenic threshold = 10 |
| DVAR | 10.0000 | 11,255 | 10,972 | 1.0258 | 0.9991 | 1.0532 | 0.0586 | Pathogenic threshold = 10 |
| Eigen_PC | 10.0000 | 13,658 | 13,269 | 1.0293 | 1.0049 | 1.0543 | 0.0181 | Pathogenic threshold = 10 |
| Eigen | 10.0000 | 11,128 | 10,820 | 1.0285 | 1.0015 | 1.0561 | 0.0382 | Pathogenic threshold = 10 |
| FATHMM-MKL | 10.0000 | 9,333 | 9,111 | 1.0244 | 0.9951 | 1.0545 | 0.1037 | Pathogenic threshold = 10 |
| FATHMM-XF | 10.0000 | 10,358 | 10,233 | 1.0122 | 0.9848 | 1.0403 | 0.3875 | Pathogenic threshold = 10 |
| FIRE | 10.0000 | 11,593 | 11,449 | 1.0126 | 0.9867 | 1.0392 | 0.3462 | Pathogenic threshold = 10 |
| fitCons | 10.0000 | 13,966 | 13,541 | 1.0314 | 1.0072 | 1.0561 | 0.0106 | Pathogenic threshold = 10 |
| FitCons2 | 10.0000 | 11,925 | 11,533 | 1.0340 | 1.0078 | 1.0609 | 0.0107 | Pathogenic threshold = 10 |
| FunSeq2 | 10.0000 | 12,185 | 11,910 | 1.0231 | 0.9975 | 1.0493 | 0.0775 | Pathogenic threshold = 10 |
| GenoCanyon | 10.0000 | 13,418 | 12,980 | 1.0337 | 1.0090 | 1.0591 | 0.0072 | Pathogenic threshold = 10 |
| LINSIGHT | 10.0000 | 12,064 | 11,655 | 1.0351 | 1.0090 | 1.0619 | 0.0081 | Pathogenic threshold = 10 |
| ncER | 10.0000 | 9,183 | 8,901 | 1.0317 | 1.0019 | 1.0623 | 0.0367 | Pathogenic threshold = 10 |
| Orion | 10.0000 | 12,205 | 11,571 | 1.0548 | 1.0282 | 1.0821 | 0.0000 | Pathogenic threshold = 10 |
| PAFA | 10.0000 | 12,090 | 11,747 | 1.0292 | 1.0033 | 1.0558 | 0.0267 | Pathogenic threshold = 10 |
| ReMM | 10.0000 | 10,136 | 9,856 | 1.0284 | 1.0002 | 1.0574 | 0.0485 | Pathogenic threshold = 10 |
| regBase_REG | 10.0000 | 12,594 | 12,363 | 1.0187 | 0.9936 | 1.0444 | 0.1454 | Pathogenic threshold = 10 |
| regBase_CAN | 10.0000 | 9,430 | 9,425 | 1.0005 | 0.9723 | 1.0296 | 0.9768 | Pathogenic threshold = 10 |
| regBase_PAT | 10.0000 | 6,460 | 6,405 | 1.0086 | 0.9742 | 1.0442 | 0.6340 | Pathogenic threshold = 10 |
| DIVAN_TSS | 10.0000 | 12,357 | 12,240 | 1.0096 | 0.9846 | 1.0352 | 0.4595 | Pathogenic threshold = 10 |
| DIVAN_REGION | 10.0000 | 11,840 | 11,770 | 1.0059 | 0.9805 | 1.0320 | 0.6534 | Pathogenic threshold = 10 |
| CADD | 15.0000 | 2,769 | 2,589 | 1.0695 | 1.0134 | 1.1288 | 0.0145 | Pathogenic threshold = 15 |
| CDTS | 15.0000 | 3,192 | 3,005 | 1.0622 | 1.0103 | 1.1169 | 0.0181 | Pathogenic threshold = 15 |
| CScape | 15.0000 | 1,957 | 1,982 | 0.9874 | 0.9271 | 1.0516 | 0.7022 | Pathogenic threshold = 15 |
| DANN | 15.0000 | 2,595 | 2,527 | 1.0269 | 0.9718 | 1.0852 | 0.3492 | Pathogenic threshold = 15 |
| DVAR | 15.0000 | 2,702 | 2,600 | 1.0392 | 0.9844 | 1.0971 | 0.1654 | Pathogenic threshold = 15 |
| Eigen_PC | 15.0000 | 4,691 | 4,516 | 1.0388 | 0.9970 | 1.0823 | 0.0698 | Pathogenic threshold = 15 |
| Eigen | 15.0000 | 3,685 | 3,566 | 1.0334 | 0.9866 | 1.0824 | 0.1658 | Pathogenic threshold = 15 |
| FATHMM-MKL | 15.0000 | 2,590 | 2,557 | 1.0129 | 0.9587 | 1.0702 | 0.6556 | Pathogenic threshold = 15 |
| FATHMM-XF | 15.0000 | 3,259 | 3,129 | 1.0415 | 0.9914 | 1.0943 | 0.1065 | Pathogenic threshold = 15 |
| FIRE | 15.0000 | 3,858 | 3,826 | 1.0084 | 0.9640 | 1.0548 | 0.7236 | Pathogenic threshold = 15 |
| fitCons | 15.0000 | 2,971 | 2,776 | 1.0702 | 1.0159 | 1.1275 | 0.0105 | Pathogenic threshold = 15 |
| FitCons2 | 15.0000 | 2,657 | 2,468 | 1.0766 | 1.0188 | 1.1377 | 0.0086 | Pathogenic threshold = 15 |
| FunSeq2 | 15.0000 | 3,933 | 3,775 | 1.0419 | 0.9961 | 1.0897 | 0.0737 | Pathogenic threshold = 15 |
| GenoCanyon | 15.0000 | 4,349 | 4,241 | 1.0255 | 0.9828 | 1.0700 | 0.2483 | Pathogenic threshold = 15 |
| LINSIGHT | 15.0000 | 3,666 | 3,463 | 1.0586 | 1.0103 | 1.1093 | 0.0167 | Pathogenic threshold = 15 |
| ncER | 15.0000 | 2,847 | 2,806 | 1.0146 | 0.9627 | 1.0693 | 0.5947 | Pathogenic threshold = 15 |
| Orion | 15.0000 | 3,604 | 3,429 | 1.0510 | 1.0027 | 1.1017 | 0.0380 | Pathogenic threshold = 15 |
| PAFA | 15.0000 | 4,132 | 3,896 | 1.0606 | 1.0149 | 1.1083 | 0.0087 | Pathogenic threshold = 15 |
| ReMM | 15.0000 | 2,837 | 2,719 | 1.0434 | 0.9896 | 1.1002 | 0.1165 | Pathogenic threshold = 15 |
| regBase_REG | 15.0000 | 4,087 | 3,947 | 1.0355 | 0.9909 | 1.0820 | 0.1210 | Pathogenic threshold = 15 |
| regBase_CAN | 15.0000 | 1,971 | 2,088 | 0.9440 | 0.8872 | 1.0044 | 0.0686 | Pathogenic threshold = 15 |
| regBase_PAT | 15.0000 | 1,548 | 1,540 | 1.0052 | 0.9361 | 1.0794 | 0.8998 | Pathogenic threshold = 15 |
| DIVAN_TSS | 15.0000 | 3,736 | 3,894 | 0.9594 | 0.9171 | 1.0037 | 0.0723 | Pathogenic threshold = 15 |
| DIVAN_REGION | 15.0000 | 3,683 | 3,611 | 1.0199 | 0.9739 | 1.0681 | 0.4058 | Pathogenic threshold = 15 |
| CADD | 20.0000 | 453 | 401 | 1.1297 | 0.9854 | 1.2954 | 0.0809 | Pathogenic threshold = 20 |
| CDTS | 20.0000 | 1,077 | 952 | 1.1313 | 1.0359 | 1.2357 | 0.0059 | Pathogenic threshold = 20 |
| CScape | 20.0000 | 598 | 667 | 0.8966 | 0.8015 | 1.0027 | 0.0558 | Pathogenic threshold = 20 |
| DANN | 20.0000 | 383 | 384 | 0.9974 | 0.8635 | 1.1521 | 1.0000 | Pathogenic threshold = 20 |
| DVAR | 20.0000 | 727 | 710 | 1.0239 | 0.9221 | 1.1371 | 0.6730 | Pathogenic threshold = 20 |
| Eigen_PC | 20.0000 | 1,595 | 1,489 | 1.0712 | 0.9975 | 1.1504 | 0.0586 | Pathogenic threshold = 20 |
| Eigen | 20.0000 | 1,224 | 1,171 | 1.0453 | 0.9640 | 1.1334 | 0.2880 | Pathogenic threshold = 20 |
| FATHMM-MKL | 20.0000 | 653 | 662 | 0.9864 | 0.8840 | 1.1007 | 0.8254 | Pathogenic threshold = 20 |
| FATHMM-XF | 20.0000 | 1,028 | 1,015 | 1.0128 | 0.9278 | 1.1057 | 0.7906 | Pathogenic threshold = 20 |
| FIRE | 20.0000 | 1,242 | 1,245 | 0.9976 | 0.9214 | 1.0800 | 0.9680 | Pathogenic threshold = 20 |
| fitCons | 20.0000 | 62 | 69 | 0.8986 | 0.6272 | 1.2849 | 0.6003 | Pathogenic threshold = 20 |
| FitCons2 | 20.0000 | 71 | 66 | 1.0758 | 0.7585 | 1.5275 | 0.7327 | Pathogenic threshold = 20 |
| FunSeq2 | 20.0000 | 1,335 | 1,335 | 1.0000 | 0.9262 | 1.0796 | 1.0000 | Pathogenic threshold = 20 |
| GenoCanyon | 20.0000 | 1,422 | 1,342 | 1.0596 | 0.9828 | 1.1425 | 0.1329 | Pathogenic threshold = 20 |
| LINSIGHT | 20.0000 | 1,102 | 1,082 | 1.0185 | 0.9357 | 1.1086 | 0.6843 | Pathogenic threshold = 20 |
| ncER | 20.0000 | 910 | 942 | 0.9660 | 0.8809 | 1.0593 | 0.4713 | Pathogenic threshold = 20 |
| Orion | 20.0000 | 1,138 | 1,019 | 1.1168 | 1.0254 | 1.2165 | 0.0110 | Pathogenic threshold = 20 |
| PAFA | 20.0000 | 1,576 | 1,444 | 1.0914 | 1.0156 | 1.1730 | 0.0171 | Pathogenic threshold = 20 |
| ReMM | 20.0000 | 727 | 689 | 1.0552 | 0.9494 | 1.1727 | 0.3255 | Pathogenic threshold = 20 |
| regBase_REG | 20.0000 | 1,331 | 1,265 | 1.0522 | 0.9735 | 1.1372 | 0.2020 | Pathogenic threshold = 20 |
| regBase_CAN | 20.0000 | 584 | 640 | 0.9125 | 0.8143 | 1.0224 | 0.1159 | Pathogenic threshold = 20 |
| regBase_PAT | 20.0000 | 482 | 479 | 1.0063 | 0.8849 | 1.1443 | 0.9486 | Pathogenic threshold = 20 |
| DIVAN_TSS | 20.0000 | 1,136 | 1,206 | 0.9420 | 0.8679 | 1.0223 | 0.1539 | Pathogenic threshold = 20 |
| DIVAN_REGION | 20.0000 | 1,132 | 1,086 | 1.0424 | 0.9582 | 1.1339 | 0.3393 | Pathogenic threshold = 20 |
| CADD | 25.0000 | 2 | 1 | 2.0000 | 0.1041 | 117.9944 | 1.0000 | Pathogenic threshold = 25 |
| CDTS | 25.0000 | 362 | 332 | 1.0904 | 0.9369 | 1.2693 | 0.2710 | Pathogenic threshold = 25 |
| CScape | 25.0000 | 211 | 231 | 0.9134 | 0.7543 | 1.1057 | 0.3662 | Pathogenic threshold = 25 |
| DANN | 25.0000 | 4 | 3 | 1.3333 | 0.2256 | 9.1022 | 1.0000 | Pathogenic threshold = 25 |
| DVAR | 25.0000 | 252 | 235 | 1.0723 | 0.8941 | 1.2864 | 0.4685 | Pathogenic threshold = 25 |
| Eigen_PC | 25.0000 | 553 | 516 | 1.0717 | 0.9488 | 1.2107 | 0.2709 | Pathogenic threshold = 25 |
| Eigen | 25.0000 | 421 | 404 | 1.0421 | 0.9069 | 1.1975 | 0.5775 | Pathogenic threshold = 25 |
| FATHMM-MKL | 25.0000 | 121 | 157 | 0.7707 | 0.6029 | 0.9832 | 0.0356 | Pathogenic threshold = 25 |
| FATHMM-XF | 25.0000 | 279 | 301 | 0.9269 | 0.7847 | 1.0945 | 0.3832 | Pathogenic threshold = 25 |
| FIRE | 25.0000 | 385 | 397 | 0.9698 | 0.8407 | 1.1186 | 0.6941 | Pathogenic threshold = 25 |
| fitCons | 25.0000 | 14 | 14 | 1.0000 | 0.4419 | 2.2630 | 1.0000 | Pathogenic threshold = 25 |
| FitCons2 | 25.0000 | 25 | 19 | 1.3158 | 0.6959 | 2.5274 | 0.4514 | Pathogenic threshold = 25 |
| FunSeq2 | 25.0000 | 466 | 454 | 1.0264 | 0.9000 | 1.1706 | 0.7169 | Pathogenic threshold = 25 |
| GenoCanyon | 25.0000 | 474 | 464 | 1.0216 | 0.8969 | 1.1636 | 0.7689 | Pathogenic threshold = 25 |
| LINSIGHT | 25.0000 | 393 | 360 | 1.0917 | 0.9438 | 1.2630 | 0.2435 | Pathogenic threshold = 25 |
| ncER | 25.0000 | 264 | 276 | 0.9565 | 0.8049 | 1.1365 | 0.6360 | Pathogenic threshold = 25 |
| Orion | 25.0000 | 332 | 303 | 1.0957 | 0.9349 | 1.2846 | 0.2665 | Pathogenic threshold = 25 |
| PAFA | 25.0000 | 844 | 782 | 1.0793 | 0.9781 | 1.1911 | 0.1303 | Pathogenic threshold = 25 |
| ReMM | 25.0000 | 244 | 214 | 1.1402 | 0.9451 | 1.3764 | 0.1753 | Pathogenic threshold = 25 |
| regBase_REG | 25.0000 | 419 | 385 | 1.0883 | 0.9454 | 1.2531 | 0.2445 | Pathogenic threshold = 25 |
| regBase_CAN | 25.0000 | 191 | 191 | 1.0000 | 0.8140 | 1.2286 | 1.0000 | Pathogenic threshold = 25 |
| regBase_PAT | 25.0000 | 138 | 133 | 1.0376 | 0.8117 | 1.3267 | 0.8081 | Pathogenic threshold = 25 |
| DIVAN_TSS | 25.0000 | 355 | 362 | 0.9807 | 0.8447 | 1.1384 | 0.8227 | Pathogenic threshold = 25 |
| DIVAN_REGION | 25.0000 | 358 | 334 | 1.0719 | 0.9208 | 1.2480 | 0.3820 | Pathogenic threshold = 25 |
| CADD | 30.0000 | 1 | 0 | 1.0000 | 0.0256 | 1.0000 | 1.0000 | Pathogenic threshold = 30 |
| CDTS | 30.0000 | 119 | 90 | 1.3222 | 0.9971 | 1.7585 | 0.0525 | Pathogenic threshold = 30 |
| CScape | 30.0000 | 76 | 83 | 0.9157 | 0.6619 | 1.2653 | 0.6343 | Pathogenic threshold = 30 |
| DANN | 30.0000 | 2 | 0 | 1.0000 | 0.1878 | 1.0000 | 0.5000 | Pathogenic threshold = 30 |
| DVAR | 30.0000 | 65 | 74 | 0.8784 | 0.6196 | 1.2426 | 0.4976 | Pathogenic threshold = 30 |
| Eigen_PC | 30.0000 | 206 | 174 | 1.1839 | 0.9628 | 1.4571 | 0.1117 | Pathogenic threshold = 30 |
| Eigen | 30.0000 | 155 | 145 | 1.0690 | 0.8468 | 1.3500 | 0.6034 | Pathogenic threshold = 30 |
| FATHMM-MKL | 30.0000 | 45 | 62 | 0.7258 | 0.4832 | 1.0827 | 0.1215 | Pathogenic threshold = 30 |
| FATHMM-XF | 30.0000 | 74 | 67 | 1.1045 | 0.7828 | 1.5607 | 0.6135 | Pathogenic threshold = 30 |
| FIRE | 30.0000 | 144 | 138 | 1.0435 | 0.8204 | 1.3276 | 0.7660 | Pathogenic threshold = 30 |
| fitCons | 30.0000 | 3 | 4 | 0.7500 | 0.1099 | 4.4333 | 1.0000 | Pathogenic threshold = 30 |
| FitCons2 | 30.0000 | 24 | 18 | 1.3333 | 0.6938 | 2.6074 | 0.4408 | Pathogenic threshold = 30 |
| FunSeq2 | 30.0000 | 170 | 137 | 1.2409 | 0.9850 | 1.5656 | 0.0676 | Pathogenic threshold = 30 |
| GenoCanyon | 30.0000 | 306 | 269 | 1.1375 | 0.9625 | 1.3451 | 0.1332 | Pathogenic threshold = 30 |
| LINSIGHT | 30.0000 | 136 | 119 | 1.1429 | 0.8870 | 1.4742 | 0.3164 | Pathogenic threshold = 30 |
| ncER | 30.0000 | 47 | 61 | 0.7705 | 0.5152 | 1.1459 | 0.2108 | Pathogenic threshold = 30 |
| Orion | 30.0000 | 97 | 96 | 1.0104 | 0.7541 | 1.3541 | 1.0000 | Pathogenic threshold = 30 |
| PAFA | 30.0000 | 844 | 782 | 1.0793 | 0.9781 | 1.1911 | 0.1303 | Pathogenic threshold = 30 |
| ReMM | 30.0000 | 84 | 69 | 1.2174 | 0.8748 | 1.6990 | 0.2576 | Pathogenic threshold = 30 |
| regBase_REG | 30.0000 | 137 | 121 | 1.1322 | 0.8801 | 1.4580 | 0.3504 | Pathogenic threshold = 30 |
| regBase_CAN | 30.0000 | 53 | 48 | 1.1042 | 0.7330 | 1.6671 | 0.6908 | Pathogenic threshold = 30 |
| regBase_PAT | 30.0000 | 63 | 64 | 0.9844 | 0.6840 | 1.4163 | 1.0000 | Pathogenic threshold = 30 |
| DIVAN_TSS | 30.0000 | 113 | 105 | 1.0762 | 0.8178 | 1.4173 | 0.6355 | Pathogenic threshold = 30 |
| DIVAN_REGION | 30.0000 | 117 | 108 | 1.0833 | 0.8268 | 1.4205 | 0.5939 | Pathogenic threshold = 30 |
| CADD | 35.0000 | 0 | 0 | 1.0000 | 0.0000 | 1.0000 | 1.0000 | Pathogenic threshold = 35 |
| CDTS | 35.0000 | 32 | 20 | 1.6000 | 0.8875 | 2.9518 | 0.1263 | Pathogenic threshold = 35 |
| CScape | 35.0000 | 11 | 13 | 0.8462 | 0.3432 | 2.0468 | 0.8388 | Pathogenic threshold = 35 |
| DANN | 35.0000 | 1 | 0 | 1.0000 | 0.0256 | 1.0000 | 1.0000 | Pathogenic threshold = 35 |
| DVAR | 35.0000 | 32 | 30 | 1.0667 | 0.6277 | 1.8171 | 0.8991 | Pathogenic threshold = 35 |
| Eigen_PC | 35.0000 | 71 | 65 | 1.0923 | 0.7692 | 1.5535 | 0.6683 | Pathogenic threshold = 35 |
| Eigen | 35.0000 | 57 | 52 | 1.0962 | 0.7394 | 1.6281 | 0.7018 | Pathogenic threshold = 35 |
| FATHMM-MKL | 35.0000 | 33 | 46 | 0.7174 | 0.4444 | 1.1465 | 0.1766 | Pathogenic threshold = 35 |
| FATHMM-XF | 35.0000 | 14 | 12 | 1.1667 | 0.5008 | 2.7612 | 0.8450 | Pathogenic threshold = 35 |
| FIRE | 35.0000 | 57 | 39 | 1.4615 | 0.9557 | 2.2560 | 0.0822 | Pathogenic threshold = 35 |
| fitCons | 35.0000 | 1 | 3 | 0.3333 | 0.0063 | 4.1514 | 0.6250 | Pathogenic threshold = 35 |
| FitCons2 | 35.0000 | 23 | 18 | 1.2778 | 0.6597 | 2.5126 | 0.5327 | Pathogenic threshold = 35 |
| FunSeq2 | 35.0000 | 45 | 44 | 1.0227 | 0.6598 | 1.5862 | 1.0000 | Pathogenic threshold = 35 |
| GenoCanyon | 35.0000 | 306 | 269 | 1.1375 | 0.9625 | 1.3451 | 0.1332 | Pathogenic threshold = 35 |
| LINSIGHT | 35.0000 | 42 | 41 | 1.0244 | 0.6501 | 1.6152 | 1.0000 | Pathogenic threshold = 35 |
| ncER | 35.0000 | 2 | 10 | 0.2000 | 0.0213 | 0.9385 | 0.0386 | Pathogenic threshold = 35 |
| Orion | 35.0000 | 20 | 27 | 0.7407 | 0.3939 | 1.3709 | 0.3817 | Pathogenic threshold = 35 |
| PAFA | 35.0000 | 844 | 782 | 1.0793 | 0.9781 | 1.1911 | 0.1303 | Pathogenic threshold = 35 |
| ReMM | 35.0000 | 25 | 25 | 1.0000 | 0.5510 | 1.8147 | 1.0000 | Pathogenic threshold = 35 |
| regBase_REG | 35.0000 | 38 | 26 | 1.4615 | 0.8645 | 2.5070 | 0.1686 | Pathogenic threshold = 35 |
| regBase_CAN | 35.0000 | 17 | 24 | 0.7083 | 0.3572 | 1.3748 | 0.3489 | Pathogenic threshold = 35 |
| regBase_PAT | 35.0000 | 34 | 34 | 1.0000 | 0.6031 | 1.6581 | 1.0000 | Pathogenic threshold = 35 |
| DIVAN_TSS | 35.0000 | 36 | 36 | 1.0000 | 0.6123 | 1.6333 | 1.0000 | Pathogenic threshold = 35 |
| DIVAN_REGION | 35.0000 | 41 | 38 | 1.0789 | 0.6769 | 1.7238 | 0.8221 | Pathogenic threshold = 35 |
| CADD | 22.2285 | 50 | 35 | 1.4286 | 0.9092 | 2.2672 | 0.1284 | Top 50 DNMs in ASD |
| CDTS | 32.7426 | 50 | 40 | 1.2500 | 0.8083 | 1.9443 | 0.3428 | Top 50 DNMs in ASD |
| CScape | 31.0251 | 50 | 59 | 0.8475 | 0.5696 | 1.2566 | 0.4437 | Top 50 DNMs in ASD |
| DANN | 21.8926 | 50 | 47 | 1.0638 | 0.7000 | 1.6192 | 0.8392 | Top 50 DNMs in ASD |
| DIVAN_REGION | 19.0293 | 51 | 42 | 1.2143 | 0.7913 | 1.8724 | 0.4069 | Top 50 DNMs in ASD |
| DIVAN_TSS | 18.8821 | 51 | 49 | 1.0408 | 0.6893 | 1.5730 | 0.9204 | Top 50 DNMs in ASD |
| DVAR | 31.3630 | 60 | 68 | 0.8824 | 0.6130 | 1.2673 | 0.5363 | Top 50 DNMs in ASD |
| Eigen | 35.9826 | 50 | 38 | 1.3158 | 0.8458 | 2.0620 | 0.2408 | Top 50 DNMs in ASD |
| Eigen_PC | 36.7700 | 50 | 48 | 1.0417 | 0.6868 | 1.5813 | 0.9196 | Top 50 DNMs in ASD |
| FATHMM-MKL | 28.7705 | 50 | 72 | 0.6944 | 0.4742 | 1.0101 | 0.0568 | Top 50 DNMs in ASD |
| FATHMM-XF | 31.8651 | 50 | 43 | 1.1628 | 0.7580 | 1.7905 | 0.5341 | Top 50 DNMs in ASD |
| FIRE | 35.9983 | 57 | 39 | 1.4615 | 0.9557 | 2.2560 | 0.0822 | Top 50 DNMs in ASD |
| fitCons | 20.4645 | 50 | 50 | 1.0000 | 0.6620 | 1.5105 | 1.0000 | Top 50 DNMs in ASD |
| FitCons2 | 20.2120 | 71 | 66 | 1.0758 | 0.7585 | 1.5275 | 0.7327 | Top 50 DNMs in ASD |
| FunSeq2 | 34.8172 | 50 | 45 | 1.1111 | 0.7278 | 1.7006 | 0.6817 | Top 50 DNMs in ASD |
| GenoCanyon | 99.3328 | 306 | 269 | 1.1375 | 0.9625 | 1.3451 | 0.1332 | Top 50 DNMs in ASD |
| LINSIGHT | 34.3931 | 50 | 45 | 1.1111 | 0.7278 | 1.7006 | 0.6817 | Top 50 DNMs in ASD |
| ncER | 29.7367 | 50 | 68 | 0.7353 | 0.4999 | 1.0751 | 0.1172 | Top 50 DNMs in ASD |
| Orion | 31.9212 | 50 | 59 | 0.8475 | 0.5696 | 1.2566 | 0.4437 | Top 50 DNMs in ASD |
| PAFA | 99.3328 | 844 | 782 | 1.0793 | 0.9781 | 1.1911 | 0.1303 | Top 50 DNMs in ASD |
| regBase_REG | 34.1327 | 50 | 39 | 1.2821 | 0.8266 | 2.0014 | 0.2891 | Top 50 DNMs in ASD |
| regBase_CAN | 30.1987 | 50 | 46 | 1.0870 | 0.7136 | 1.6589 | 0.7596 | Top 50 DNMs in ASD |
| regBase_PAT | 31.2330 | 50 | 53 | 0.9434 | 0.6280 | 1.4153 | 0.8439 | Top 50 DNMs in ASD |
| ReMM | 34.4589 | 53 | 39 | 1.3590 | 0.8819 | 2.1106 | 0.1750 | Top 50 DNMs in ASD |
| CADD | 21.8455 | 100 | 76 | 1.3158 | 0.9667 | 1.7971 | 0.0827 | Top 100 DNMs in ASD |
| CDTS | 30.4395 | 100 | 79 | 1.2658 | 0.9330 | 1.7224 | 0.1347 | Top 100 DNMs in ASD |
| CScape | 29.5308 | 100 | 97 | 1.0309 | 0.7718 | 1.3775 | 0.8867 | Top 100 DNMs in ASD |
| DANN | 21.5279 | 100 | 105 | 0.9524 | 0.7169 | 1.2645 | 0.7800 | Top 100 DNMs in ASD |
| DIVAN_REGION | 30.9855 | 101 | 89 | 1.1348 | 0.8450 | 1.5264 | 0.4249 | Top 100 DNMs in ASD |
| DIVAN_TSS | 30.9182 | 100 | 81 | 1.2346 | 0.9118 | 1.6759 | 0.1808 | Top 100 DNMs in ASD |
| DVAR | 28.7393 | 106 | 110 | 0.9636 | 0.7310 | 1.2699 | 0.8383 | Top 100 DNMs in ASD |
| Eigen | 32.3566 | 100 | 93 | 1.0753 | 0.8026 | 1.4418 | 0.6659 | Top 100 DNMs in ASD |
| Eigen_PC | 33.4089 | 100 | 95 | 1.0526 | 0.7869 | 1.4089 | 0.7746 | Top 100 DNMs in ASD |
| FATHMM-MKL | 25.5653 | 100 | 131 | 0.7634 | 0.5825 | 0.9979 | 0.0482 | Top 100 DNMs in ASD |
| FATHMM-XF | 28.9296 | 100 | 96 | 1.0417 | 0.7793 | 1.3930 | 0.8304 | Top 100 DNMs in ASD |
| FIRE | 32.5824 | 100 | 77 | 1.2987 | 0.9552 | 1.7715 | 0.0979 | Top 100 DNMs in ASD |
| fitCons | 19.2303 | 120 | 115 | 1.0435 | 0.8012 | 1.3595 | 0.7942 | Top 100 DNMs in ASD |
| FitCons2 | 19.5952 | 119 | 119 | 1.0000 | 0.7690 | 1.3003 | 1.0000 | Top 100 DNMs in ASD |
| FunSeq2 | 32.3758 | 100 | 75 | 1.3333 | 0.9785 | 1.8235 | 0.0693 | Top 100 DNMs in ASD |
| GenoCanyon | 99.3328 | 306 | 269 | 1.1375 | 0.9625 | 1.3451 | 0.1332 | Top 100 DNMs in ASD |
| LINSIGHT | 31.2790 | 100 | 90 | 1.1111 | 0.8274 | 1.4940 | 0.5139 | Top 100 DNMs in ASD |
| ncER | 28.1597 | 100 | 112 | 0.8929 | 0.6749 | 1.1798 | 0.4500 | Top 100 DNMs in ASD |
| Orion | 29.8353 | 100 | 101 | 0.9901 | 0.7433 | 1.3186 | 1.0000 | Top 100 DNMs in ASD |
| PAFA | 99.3328 | 844 | 782 | 1.0793 | 0.9781 | 1.1911 | 0.1303 | Top 100 DNMs in ASD |
| regBase_REG | 31.4782 | 100 | 85 | 1.1765 | 0.8722 | 1.5899 | 0.3033 | Top 100 DNMs in ASD |
| regBase_CAN | 27.6023 | 100 | 81 | 1.2346 | 0.9118 | 1.6759 | 0.1808 | Top 100 DNMs in ASD |
| regBase_PAT | 26.7619 | 100 | 98 | 1.0204 | 0.7645 | 1.3623 | 0.9434 | Top 100 DNMs in ASD |
| ReMM | 29.0541 | 132 | 106 | 1.2453 | 0.9571 | 1.6235 | 0.1049 | Top 100 DNMs in ASD |
| CADD | 21.6241 | 150 | 100 | 1.5000 | 1.1569 | 1.9517 | 0.0019 | Top 150 DNMs in ASD |
| CDTS | 28.9781 | 150 | 123 | 1.2195 | 0.9544 | 1.5607 | 0.1154 | Top 150 DNMs in ASD |
| CScape | 27.6840 | 150 | 151 | 0.9934 | 0.7872 | 1.2536 | 1.0000 | Top 150 DNMs in ASD |
| DANN | 21.1465 | 150 | 167 | 0.8982 | 0.7156 | 1.1266 | 0.3689 | Top 150 DNMs in ASD |
| DIVAN_REGION | 28.4792 | 151 | 153 | 0.9869 | 0.7830 | 1.2439 | 0.9543 | Top 150 DNMs in ASD |
| DIVAN_TSS | 28.8467 | 152 | 146 | 1.0411 | 0.8241 | 1.3156 | 0.7721 | Top 150 DNMs in ASD |
| DVAR | 26.8063 | 167 | 162 | 1.0309 | 0.8255 | 1.2876 | 0.8255 | Top 150 DNMs in ASD |
| Eigen | 30.2956 | 150 | 131 | 1.1450 | 0.8997 | 1.4587 | 0.2829 | Top 150 DNMs in ASD |
| Eigen_PC | 31.5039 | 150 | 127 | 1.1811 | 0.9263 | 1.5080 | 0.1861 | Top 150 DNMs in ASD |
| FATHMM-MKL | 24.6066 | 151 | 179 | 0.8436 | 0.6748 | 1.0535 | 0.1371 | Top 150 DNMs in ASD |
| FATHMM-XF | 27.4740 | 150 | 140 | 1.0714 | 0.8453 | 1.3587 | 0.5972 | Top 150 DNMs in ASD |
| FIRE | 29.6693 | 160 | 161 | 0.9938 | 0.7935 | 1.2446 | 1.0000 | Top 150 DNMs in ASD |
| fitCons | 19.1281 | 306 | 280 | 1.0929 | 0.9263 | 1.2898 | 0.3017 | Top 150 DNMs in ASD |
| FitCons2 | 19.4405 | 372 | 342 | 1.0877 | 0.9366 | 1.2635 | 0.2778 | Top 150 DNMs in ASD |
| FunSeq2 | 30.5943 | 150 | 121 | 1.2397 | 0.9691 | 1.5884 | 0.0888 | Top 150 DNMs in ASD |
| GenoCanyon | 99.3328 | 306 | 269 | 1.1375 | 0.9625 | 1.3451 | 0.1332 | Top 150 DNMs in ASD |
| LINSIGHT | 29.5931 | 150 | 132 | 1.1364 | 0.8933 | 1.4469 | 0.3114 | Top 150 DNMs in ASD |
| ncER | 26.6932 | 150 | 176 | 0.8523 | 0.6809 | 1.0657 | 0.1661 | Top 150 DNMs in ASD |
| Orion | 28.3423 | 150 | 143 | 1.0490 | 0.8286 | 1.3284 | 0.7260 | Top 150 DNMs in ASD |
| PAFA | 99.3328 | 844 | 782 | 1.0793 | 0.9781 | 1.1911 | 0.1303 | Top 150 DNMs in ASD |
| regBase_REG | 29.5488 | 150 | 137 | 1.0949 | 0.8627 | 1.3905 | 0.4788 | Top 150 DNMs in ASD |
| regBase_CAN | 25.6810 | 150 | 152 | 0.9868 | 0.7823 | 1.2448 | 0.9541 | Top 150 DNMs in ASD |
| regBase_PAT | 24.6028 | 150 | 144 | 1.0417 | 0.8232 | 1.3185 | 0.7706 | Top 150 DNMs in ASD |
| ReMM | 27.4452 | 169 | 140 | 1.2071 | 0.9592 | 1.5212 | 0.1110 | Top 150 DNMs in ASD |
| CADD | 21.2419 | 200 | 149 | 1.3423 | 1.0803 | 1.6708 | 0.0074 | Top 200 DNMs in ASD |
| CDTS | 27.9946 | 200 | 165 | 1.2121 | 0.9814 | 1.4988 | 0.0750 | Top 200 DNMs in ASD |
| CScape | 25.2729 | 200 | 223 | 0.8969 | 0.7373 | 1.0904 | 0.2848 | Top 200 DNMs in ASD |
| DANN | 20.8830 | 200 | 214 | 0.9346 | 0.7668 | 1.1386 | 0.5229 | Top 200 DNMs in ASD |
| DIVAN_REGION | 27.3773 | 203 | 205 | 0.9902 | 0.8115 | 1.2083 | 0.9605 | Top 200 DNMs in ASD |
| DIVAN_TSS | 27.4981 | 200 | 195 | 1.0256 | 0.8378 | 1.2558 | 0.8405 | Top 200 DNMs in ASD |
| DVAR | 25.9737 | 205 | 191 | 1.0733 | 0.8770 | 1.3141 | 0.5136 | Top 200 DNMs in ASD |
| Eigen | 28.5822 | 200 | 194 | 1.0309 | 0.8419 | 1.2626 | 0.8012 | Top 200 DNMs in ASD |
| Eigen_PC | 30.1946 | 200 | 165 | 1.2121 | 0.9814 | 1.4988 | 0.0750 | Top 200 DNMs in ASD |
| FATHMM-MKL | 23.7084 | 200 | 245 | 0.8163 | 0.6738 | 0.9880 | 0.0369 | Top 200 DNMs in ASD |
| FATHMM-XF | 26.2866 | 200 | 202 | 0.9901 | 0.8102 | 1.2099 | 0.9602 | Top 200 DNMs in ASD |
| FIRE | 28.5879 | 204 | 205 | 0.9951 | 0.8157 | 1.2139 | 1.0000 | Top 200 DNMs in ASD |
| fitCons | 19.1281 | 306 | 280 | 1.0929 | 0.9263 | 1.2898 | 0.3017 | Top 200 DNMs in ASD |
| FitCons2 | 19.4405 | 372 | 342 | 1.0877 | 0.9366 | 1.2635 | 0.2778 | Top 200 DNMs in ASD |
| FunSeq2 | 29.2104 | 201 | 177 | 1.1356 | 0.9232 | 1.3978 | 0.2368 | Top 200 DNMs in ASD |
| GenoCanyon | 99.3328 | 306 | 269 | 1.1375 | 0.9625 | 1.3451 | 0.1332 | Top 200 DNMs in ASD |
| LINSIGHT | 28.1606 | 200 | 187 | 1.0695 | 0.8718 | 1.3126 | 0.5419 | Top 200 DNMs in ASD |
| ncER | 25.9870 | 200 | 208 | 0.9615 | 0.7879 | 1.1732 | 0.7290 | Top 200 DNMs in ASD |
| Orion | 27.2464 | 200 | 184 | 1.0870 | 0.8853 | 1.3352 | 0.4440 | Top 200 DNMs in ASD |
| PAFA | 99.3328 | 844 | 782 | 1.0793 | 0.9781 | 1.1911 | 0.1303 | Top 200 DNMs in ASD |
| regBase_REG | 28.1902 | 200 | 181 | 1.1050 | 0.8992 | 1.3586 | 0.3565 | Top 200 DNMs in ASD |
| regBase_CAN | 24.7373 | 200 | 202 | 0.9901 | 0.8102 | 1.2099 | 0.9602 | Top 200 DNMs in ASD |
| regBase_PAT | 23.1036 | 200 | 198 | 1.0101 | 0.8257 | 1.2357 | 0.9600 | Top 200 DNMs in ASD |
| ReMM | 26.2316 | 211 | 178 | 1.1854 | 0.9664 | 1.4553 | 0.1046 | Top 200 DNMs in ASD |
| CADD | 21.0103 | 250 | 192 | 1.3021 | 1.0745 | 1.5798 | 0.0066 | Top 250 DNMs in ASD |
| CDTS | 26.8855 | 250 | 207 | 1.2077 | 1.0005 | 1.4591 | 0.0493 | Top 250 DNMs in ASD |
| CScape | 24.1744 | 250 | 273 | 0.9158 | 0.7683 | 1.0912 | 0.3361 | Top 250 DNMs in ASD |
| DANN | 20.6242 | 250 | 270 | 0.9259 | 0.7764 | 1.1038 | 0.4048 | Top 250 DNMs in ASD |
| DIVAN_REGION | 26.5827 | 252 | 234 | 1.0769 | 0.8978 | 1.2922 | 0.4407 | Top 250 DNMs in ASD |
| DIVAN_TSS | 26.3557 | 252 | 253 | 0.9960 | 0.8333 | 1.1906 | 1.0000 | Top 250 DNMs in ASD |
| DVAR | 25.0378 | 252 | 235 | 1.0723 | 0.8941 | 1.2864 | 0.4685 | Top 250 DNMs in ASD |
| Eigen | 27.6044 | 250 | 240 | 1.0417 | 0.8691 | 1.2488 | 0.6844 | Top 250 DNMs in ASD |
| Eigen_PC | 29.0694 | 250 | 220 | 1.1364 | 0.9442 | 1.3684 | 0.1809 | Top 250 DNMs in ASD |
| FATHMM-MKL | 23.1645 | 250 | 284 | 0.8803 | 0.7397 | 1.0471 | 0.1532 | Top 250 DNMs in ASD |
| FATHMM-XF | 25.4267 | 250 | 265 | 0.9434 | 0.7905 | 1.1256 | 0.5373 | Top 250 DNMs in ASD |
| FIRE | 27.2538 | 261 | 255 | 1.0235 | 0.8580 | 1.2211 | 0.8258 | Top 250 DNMs in ASD |
| fitCons | 19.1281 | 306 | 280 | 1.0929 | 0.9263 | 1.2898 | 0.3017 | Top 250 DNMs in ASD |
| FitCons2 | 19.4405 | 372 | 342 | 1.0877 | 0.9366 | 1.2635 | 0.2778 | Top 250 DNMs in ASD |
| FunSeq2 | 28.1725 | 250 | 237 | 1.0549 | 0.8796 | 1.2654 | 0.5866 | Top 250 DNMs in ASD |
| GenoCanyon | 99.3328 | 306 | 269 | 1.1375 | 0.9625 | 1.3451 | 0.1332 | Top 250 DNMs in ASD |
| LINSIGHT | 26.9984 | 250 | 242 | 1.0331 | 0.8622 | 1.2379 | 0.7524 | Top 250 DNMs in ASD |
| ncER | 25.2497 | 250 | 258 | 0.9690 | 0.8110 | 1.1576 | 0.7562 | Top 250 DNMs in ASD |
| Orion | 26.4218 | 250 | 216 | 1.1574 | 0.9609 | 1.3951 | 0.1262 | Top 250 DNMs in ASD |
| PAFA | 99.3328 | 844 | 782 | 1.0793 | 0.9781 | 1.1911 | 0.1303 | Top 250 DNMs in ASD |
| regBase_REG | 27.2604 | 250 | 220 | 1.1364 | 0.9442 | 1.3684 | 0.1809 | Top 250 DNMs in ASD |
| regBase_CAN | 23.7142 | 250 | 268 | 0.9328 | 0.7820 | 1.1125 | 0.4551 | Top 250 DNMs in ASD |
| regBase_PAT | 22.1223 | 250 | 258 | 0.9690 | 0.8110 | 1.1576 | 0.7562 | Top 250 DNMs in ASD |
| ReMM | 24.5162 | 281 | 249 | 1.1285 | 0.9481 | 1.3438 | 0.1781 | Top 250 DNMs in ASD |
| CADD | 20.6685 | 300 | 258 | 1.1628 | 0.9812 | 1.3787 | 0.0825 | Top 300 DNMs in ASD |
| CDTS | 26.1352 | 300 | 254 | 1.1811 | 0.9960 | 1.4015 | 0.0558 | Top 300 DNMs in ASD |
| CScape | 23.3983 | 300 | 322 | 0.9317 | 0.7934 | 1.0938 | 0.3998 | Top 300 DNMs in ASD |
| DANN | 20.3789 | 300 | 312 | 0.9615 | 0.8179 | 1.1303 | 0.6566 | Top 300 DNMs in ASD |
| DIVAN_REGION | 25.7736 | 305 | 281 | 1.0854 | 0.9200 | 1.2810 | 0.3421 | Top 300 DNMs in ASD |
| DIVAN_TSS | 25.6030 | 300 | 312 | 0.9615 | 0.8179 | 1.1303 | 0.6566 | Top 300 DNMs in ASD |
| DVAR | 24.1092 | 308 | 300 | 1.0267 | 0.8729 | 1.2076 | 0.7765 | Top 300 DNMs in ASD |
| Eigen | 26.5387 | 300 | 310 | 0.9677 | 0.8229 | 1.1379 | 0.7156 | Top 300 DNMs in ASD |
| Eigen_PC | 27.9394 | 300 | 275 | 1.0909 | 0.9231 | 1.2896 | 0.3169 | Top 300 DNMs in ASD |
| FATHMM-MKL | 22.6063 | 300 | 332 | 0.9036 | 0.7704 | 1.0595 | 0.2175 | Top 300 DNMs in ASD |
| FATHMM-XF | 24.6196 | 300 | 333 | 0.9009 | 0.7682 | 1.0562 | 0.2034 | Top 300 DNMs in ASD |
| FIRE | 26.5044 | 310 | 297 | 1.0438 | 0.8873 | 1.2280 | 0.6262 | Top 300 DNMs in ASD |
| fitCons | 19.1281 | 306 | 280 | 1.0929 | 0.9263 | 1.2898 | 0.3017 | Top 300 DNMs in ASD |
| FitCons2 | 19.4405 | 372 | 342 | 1.0877 | 0.9366 | 1.2635 | 0.2778 | Top 300 DNMs in ASD |
| FunSeq2 | 27.2648 | 300 | 281 | 1.0676 | 0.9043 | 1.2608 | 0.4552 | Top 300 DNMs in ASD |
| GenoCanyon | 99.3328 | 306 | 269 | 1.1375 | 0.9625 | 1.3451 | 0.1332 | Top 300 DNMs in ASD |
| LINSIGHT | 26.2336 | 300 | 279 | 1.0753 | 0.9105 | 1.2702 | 0.4059 | Top 300 DNMs in ASD |
| ncER | 24.6434 | 300 | 301 | 0.9967 | 0.8466 | 1.1734 | 1.0000 | Top 300 DNMs in ASD |
| Orion | 25.5904 | 300 | 267 | 1.1236 | 0.9496 | 1.3300 | 0.1789 | Top 300 DNMs in ASD |
| PAFA | 99.3328 | 844 | 782 | 1.0793 | 0.9781 | 1.1911 | 0.1303 | Top 300 DNMs in ASD |
| regBase_REG | 26.4005 | 300 | 278 | 1.0791 | 0.9136 | 1.2750 | 0.3824 | Top 300 DNMs in ASD |
| regBase_CAN | 22.9110 | 300 | 332 | 0.9036 | 0.7704 | 1.0595 | 0.2175 | Top 300 DNMs in ASD |
| regBase_PAT | 21.6681 | 300 | 302 | 0.9934 | 0.8439 | 1.1694 | 0.9675 | Top 300 DNMs in ASD |
| ReMM | 23.8832 | 322 | 277 | 1.1625 | 0.9869 | 1.3700 | 0.0721 | Top 300 DNMs in ASD |

*Note*: The OR, 95% confidence interval, and *P* values were calculated by Poisson’s ratio test. DNM, *de novo* mutation; ASD, autism spectrum disorder; OR, odd ratio; CI, confidential interval.
