# Supplementary material for: Performance Comparison of Computational Methods for the Prediction of the Function and Pathogenicity of Non-coding Variants
Source: Genomics Proteomics Bioinformatics. 2022 Mar 8;21(3):649–61. doi: 10.1016/j.gpb.2022.02.002 (PMC10787016; doi:10.1016/j.gpb.2022.02.002)
Supplement: Supplementary Table S7 [file mmc7.docx]

**Table S7** **Performance of 24 methods based on 4 benchmark datasets**

| Method | Germline | Somatic | eQTL | GWAS |
| --- | --- | --- | --- | --- |
| CADD | I | III | II | I |
| CDTS | III | II | III | I |
| CScape | II | II | III | III |
| DANN | II | III | III | II |
| DIVAN_REGION | III | III | I | I |
| DIVAN_TSS | III | III | I | I |
| DVAR | II | I | II | II |
| Eigen | I | II | I | II |
| Eigen_PC | III | I | I | II |
| FATHMM-MKL | I | II | II | III |
| FATHMM-XF | I | III | III | III |
| FIRE | III | II | I | II |
| fitCons | III | II | II | II |
| FitCons2 | II | I | I | III |
| FunSeq2 | II | I | II | II |
| GenoCanyon | III | I | II | II |
| LINSIGHT | I | I | I | I |
| ncER | I | I | II | III |
| Orion | III | III | III | I |
| PAFA | II | III | II | I |
| regBase_CAN | II | I | III | III |
| regBase_PAT | I | II | III | III |
| regBase_REG | II | II | I | I |
| ReMM | I | III | III | III |

*Note*: We divided 24 methods into three groups (I, II, and III) based on the rank of AUC values and every group contained 8 methods. Germline represent the first benchmark dataset (rare germline variants from ClinVar); Somatic represent the second benchmark dataset (rare somatic variants from COSMIC); eQTL represent the third benchmark dataset (common regulatory variants from curated eQTL data); GWAS represent the fourth benchmark dataset (disease-associated common variants from curated GWAS).
