# Supplementary material for: Performance Comparison of Computational Methods for the Prediction of the Function and Pathogenicity of Non-coding Variants
Source: Genomics Proteomics Bioinformatics. 2022 Mar 8;21(3):649–61. doi: 10.1016/j.gpb.2022.02.002 (PMC10787016; doi:10.1016/j.gpb.2022.02.002)
Supplement: Supplementary Table S8 [file mmc8.docx]

**Table S8 Performance evaluation based on experimentally validated *de novo* mutations from ASD**

| Methods | Missing rate (%) | Best-threshold | PPV (%) | NPV (%) | FNR (%) | Sensitivity (%) | FPR (%) | Specificity (%) | Accuracy (%) | MCC | AUC | hspr-AUC | hser-AUC | Prediction model |
| --- | --- | --- | --- | --- | --- | --- | --- | --- | --- | --- | --- | --- | --- | --- |
| CADD | 0.00 | 6.7969 | 77.27 | 63.49 | 40.35 | 59.65 | 20.00 | 80.00 | 69.16 | 0.4020 | 0.6814 | 0.5771 | NA | SM |
| CScape | 3.74 | 2.5036 | 55.22 | 55.56 | 30.19 | 69.81 | 60.00 | 40.00 | 55.34 | 0.1028 | 0.4898 | 0.5298 | NA | SM |
| DANN | 0.00 | 11.4002 | **88.89** | 53.93 | 71.93 | 28.07 | **4.00** | **96.00** | 59.81 | 0.3210 | 0.6018 | 0.5879 | NA | SM |
| DIVAN_REGION | 0.00 | 1.8501 | 56.84 | **75.00** | **5.26** | **94.74** | 82.00 | 18.00 | 58.88 | 0.2014 | 0.4351 | NA | 0.5077 | SM |
| DIVAN_TSS | 0.00 | 1.8426 | 55.00 | 71.43 | **3.51** | **96.49** | 90.00 | 10.00 | 56.07 | 0.1310 | 0.3816 | NA | 0.5205 | SM |
| FATHMM-MKL | 0.00 | 7.5595 | 77.27 | 63.49 | 40.35 | 59.65 | 20.00 | 80.00 | 69.16 | 0.4020 | 0.6411 | 0.5502 | 0.5077 | SM |
| FATHMM-XF | 3.74 | 16.2483 | **100.00** | 52.63 | 84.91 | 15.09 | **0.00** | **100.00** | 56.31 | 0.2819 | 0.5270 | 0.5704 | NA | SM |
| FIRE | 0.00 | 3.0552 | 64.47 | **74.19** | **14.04** | **85.96** | 54.00 | 46.00 | 67.29 | 0.3516 | 0.6214 | 0.5250 | 0.5307 | SM |
| ncER | 3.74 | 8.8669 | 84.85 | 61.43 | 49.09 | 50.91 | 10.42 | 89.58 | 68.93 | 0.4329 | 0.7045 | 0.6169 | 0.5396 | SM |
| PAFA | 0.93 | 9.4311 | 67.65 | 54.17 | 58.93 | 41.07 | 22.00 | 78.00 | 58.49 | 0.2040 | 0.5248 | 0.5165 | NA | SM |
| regBase_CAN | 0.00 | 5.5775 | 75.47 | 68.52 | 29.82 | 70.18 | 26.00 | 74.00 | 71.96 | 0.4408 | **0.7775** | 0.5250 | **0.6281** | SM |
| regBase_PAT | 0.00 | 5.3423 | 66.15 | 66.67 | 24.56 | 75.44 | 44.00 | 56.00 | 66.36 | 0.3212 | 0.6926 | 0.6185 | NA | SM |
| regBase_REG | 0.00 | 13.2127 | 81.08 | 61.43 | 47.37 | 52.63 | 14.00 | 86.00 | 68.22 | 0.4052 | 0.7032 | 0.5430 | 0.5230 | SM |
| ReMM | 0.00 | 9.3627 | 75.00 | 61.90 | 42.11 | 57.89 | 22.00 | 78.00 | 67.29 | 0.3640 | 0.7049 | 0.6185 | 0.5018 | SM |
| CDTS | 19.63 | 19.5957 | **100.00** | 61.76 | 59.09 | 40.91 | **0.00** | **100.00** | 69.77 | **0.5027** | 0.6894 | **0.6970** | NA | UM |
| DVAR | 0.00 | 11.7224 | 82.98 | 70.00 | 31.58 | 68.42 | 16.00 | 84.00 | **75.70** | **0.5270** | **0.7970** | **0.6293** | **0.5989** | UM |
| Eigen | 3.74 | 7.8175 | 70.59 | 67.31 | 32.08 | 67.92 | 30.00 | 70.00 | 68.93 | 0.3791 | 0.6743 | **0.6284** | NA | UM |
| Eigen_PC | 3.74 | 12.5576 | 72.34 | 66.07 | 35.85 | 64.15 | 26.00 | 74.00 | 68.93 | 0.3828 | 0.7238 | 0.5491 | NA | UM |
| GenoCanyon | 0.00 | 11.4839 | 79.59 | 68.97 | 31.58 | 68.42 | 20.00 | 80.00 | **73.83** | 0.4849 | 0.7454 | 0.5358 | 0.5485 | UM |
| Orion | 2.80 | 7.5773 | 62.00 | 57.41 | 42.59 | 57.41 | 38.00 | 62.00 | 59.62 | 0.1941 | 0.5641 | 0.5005 | 0.5077 | UM |
| fitCons | 0.93 | 10.3880 | 73.81 | 60.94 | 44.64 | 55.36 | 22.00 | 78.00 | 66.04 | 0.3404 | 0.6159 | 0.5147 | NA | SSM |
| FitCons2 | 0.93 | 11.4394 | 85.37 | 67.69 | 37.50 | 62.50 | 12.00 | 88.00 | **74.53** | **0.5176** | **0.7721** | 0.5513 | **0.5664** | SSM |
| FunSeq2 | 2.80 | 6.6337 | 67.92 | 64.71 | 33.33 | 66.67 | 34.00 | 66.00 | 66.35 | 0.3265 | 0.6811 | 0.5328 | NA | SSM |
| LINSIGHT | 2.80 | 8.0487 | 69.35 | **73.81** | 20.37 | 79.63 | 38.00 | 62.00 | 71.15 | 0.4239 | 0.7448 | 0.5537 | 0.5442 | SSM |

*Note*: Best-threshold, the threshold corresponding to the best sum of sensitivity and specificity; PPV, positive predictive value; NPV, negative predictive value; FPR, false positive rate; FNR, false negative rate; MCC, mathew correlation coefficient; AUC, area under the curve; hspr-AUC, high-specificity regional area under the curve; hser-AUC, high-sensitivity regional area under the curve; NA, not available; SM, supervised model; UM, unsupervised model; SSM, semi-supervised model. Top three methods of every measure are represented by bold text.
