# Supplementary material for: Performance Comparison of Computational Methods for the Prediction of the Function and Pathogenicity of Non-coding Variants
Source: Genomics Proteomics Bioinformatics. 2022 Mar 8;21(3):649–61. doi: 10.1016/j.gpb.2022.02.002 (PMC10787016; doi:10.1016/j.gpb.2022.02.002)
Supplement: Supplementary Table S9 [file mmc9.docx]

**Table S9 Matched configurations of vSampler**

| Matched criteria | Configuration |
| --- | --- |
| Minor allele frequency deviation | −0.01, 0.01 |
| Distance to closest transcription start site deviation | −5 kb, 5 kb |
| Gene density in distance | 100 kb |
| Gene density deviation | −5, 5 |
| Linkage disequilibrium threshold | r^2^ > 0.8 |
| Number of variants in linkage disequilibrium deviation | −5, 5 |
